# Proteo-metabolomic investigation of transgenic rice unravels metabolic alterations and accumulation of novel proteins potentially involved in defence against *Rhizoctonia solani*

**DOI:** 10.1038/s41598-019-46885-3

**Published:** 2019-07-18

**Authors:** Subhasis Karmakar, Karabi Datta, Kutubuddin Ali Molla, Dipak Gayen, Kaushik Das, Sailendra Nath Sarkar, Swapan K. Datta

**Affiliations:** 1 Laboratory of Translational Research on Transgenic Crops, Department of Botany, University of Calcutta, 35 Ballygunge Circular Road, Kolkata, 700019 West Bengal India; 2 ICAR-National Rice Research Institute, Cuttack, 753 006 Odisha India; 3 The Huck Institute of the Life Sciences and Department of Plant Pathology and Environmental Microbiology, The Pennsylvania State University, University Park, PA-16802 USA; 4 Section of Plant Biology, School of Integrative Plant Sciences (SIPS), Cornell University, Ithaca, NY 14853 USA

**Keywords:** Plant sciences, Plant stress responses, Plant molecular biology

## Abstract

The generation of sheath blight (ShB)-resistant transgenic rice plants through the expression of *Arabidopsis* *NPR1* gene is a significant development for research in the field of biotic stress. However, to our knowledge, regulation of the proteomic and metabolic networks in the ShB-resistant transgenic rice plants has not been studied. In the present investigation, the relative proteome and metabolome profiles of the non–transformed wild-type and the *AtNPR1-*transgenic rice lines prior to and subsequent to the *R. solani* infection were investigated. Total proteins from wild type and transgenic plants were investigated using two-dimensional gel electrophoresis (2-DE) followed by mass spectrometry (MS). The metabolomics study indicated an increased abundance of various metabolites, which draws parallels with the proteomic analysis. Furthermore, the proteome data was cross-examined using network analysis which identified modules that were rich in known as well as novel immunity-related prognostic proteins, particularly the mitogen-activated protein kinase 6, probable protein phosphatase 2C1, probable trehalose-phosphate phosphatase 2 and heat shock protein. A novel protein, 14–3–3GF14f was observed to be upregulated in the leaves of the transgenic rice plants after ShB infection, and the possible mechanistic role of this protein in ShB resistance may be investigated further.

## Introduction

Rice sheath blight (ShB) is the second most-devastating and widespread disease reported from almost all the rice-cultivating nations, with an estimated annual yield loss of up to 50%^1,2^. There is absence of any variation at the genetic level known to provide resistance to ShB, causing it to be further challenging to control this disease. The causal agent for ShB disease is the basidiomycetous fungus *Rhizoctonia solani* AG1–1A. *R. solani* is an enigmatic pathogen, which possesses the ability to infect almost all the major crop plants.

Plants have developed a sophisticated network for defence against myriad of pathogens, and this network involve a variety of proteins and other organic molecules that function in a number of signal transduction pathways either prior to the infection or during the infestation^3^. Several *PR* genes, such as *chitinase* (*Chi11* and *RC7*), *thaumatin-like protein* (TLP-D34), and the defence genes, such as rice *oxalate oxidase 4* (*Osoxo4*), have been demonstrated to enhance the protection against several plant pathogens including the rice ShB fungus^3–9^. Previous studies have demonstrated that the *Arabidopsis NPR1* (a non-expresser of *PR* genes) gene serves as a key regulator in salicylic acid (SA) signalling pathway, leading to systemic acquired resistance (SAR)^10–12^. The binding of SA to NPR1 converts the NPR1 protein into a functional co-activator for the activation of several genes involved in the defence-inducing pathways^13^. The *NPR1* gene from *Arabidopsis thaliana* has been demonstrated to provide protection against various types of pathogens in different plants, such as *Arabidopsis*^14^, cotton^15^, wheat^16^, carrot^17^, tomato^18^, and rice^19–21^. Our previous studies in rice variety Pusa Sugandh-2 and Jaldi-13 have showed the effectivity of *AtNPR1* in enhancing ShB disease resistance^20,21^. These studies demonstrated the effectiveness of the transgenic expression of the *AtNPR1* gene in controlling broad spectrum pathogens. However, no study related to differential changes in the protein and metabolite expressions in the *AtNPR1*-transgenic and non-transformed wild-type plants in response to the *R solani* infection have been reported to date.

Proteomic analysis provides valuable information on the expression profile of various proteins^22^. Research on rice proteomics has progressed considerably, including the availability of functional information on the proteins in response to the various abiotic and biotic stresses^23^. One of the major events occurring in the plants during any pathogen attack or infestation is the metabolic alterations. Numerous metabolomics and proteomics studies have been performed using various plant-pathogen systems^24–26^. The comparative proteomics and metabolomics approach in the leaves of transgenic sheath blight-resistant rice may assist in exploring the novel insights into the proteome and metabolome profiles of the transgenic plants against the sheath blight disease.

In the present investigation, the rice plants that were overexpressing the *AtNPR1* gene exhibited a significant level of resistance against *R. solani*. In order to understand the underlying mechanism of resistance of these transgenic rice plants, it was decided to study protein and metabolic profile that are altered in response to the *R solani* infection. In the current study, transgenic and non-transformed wild-type (WT) plants were challenged with *R solani*, followed by proteomic and metabolomic analysis. The proteins were showed differentially upregulation and down-regulation in the transgenic rice leaves were isolated through two-dimensional gel electrophoresis (2-DE) and analysed using the matrix-assisted laser desorption ionisation-time of flight tandem mass spectrometry (MALDI-TOF-MS/MS). The GC-MS and proteomic analyses delineated a cascade of events associated with the resistance mechanism against *Rhizoctonia solani* in the transgenic rice. The present study provides insights into the rice-*R. solani* interaction and a basic understanding of the proteomic and metabolomic alternations in the transgenic rice plants in order to confer enhanced resistance.

## Results and Discussion

### Morphological variations in rice plants post sheath blight disease

**Figure 1:**
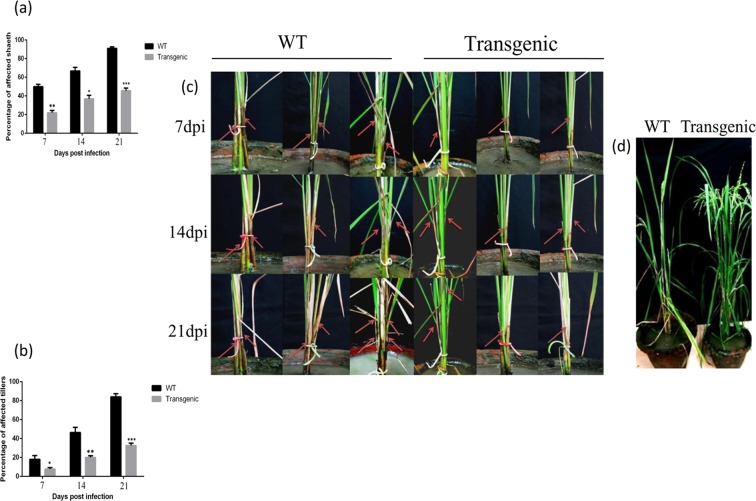
Pathogen inoculation assay and morphological alternations of two sets of three representative plants [3 plants from WT (Wild type) and 3 progeny plants from homozygous transgenic 105-5 rice line]. (**a**) Bar diagram showing percentage of affected sheath in transgenic and wild-type (WT) rice plants. Data represent means ± SE calculated from three biological replicates. (**b**) Bar diagram showing percentage of affected tillers in transgenic and wild-type (WT) plants. Data represent means ± SE calculated from three biological replicates. (**c**) Images showing sheath blight disease progression in non-transformed WT and transgenic plants at 7, 14 and 21 dpi (days post inoculation). Red arrows indicate sheath blight symptoms. (**d**) Image showing phenotype of transgenic and wild type (WT) control rice plants after 30 dpi (days post infection).

In the present investigation, *AtNPR1* expression in the transgenic rice leads to enhanced resistance against the Sheath blight (ShB) disease (Supplementary Fig. S2). The distinguishable morphological alternations between the non-transformed WT and transgenic rice plants are depicted (Fig. 1). The symptom development in WT plants began to post 72 h of ShB infection and became further pronounced post seven days. On the contrary, the number of ShB lesions formed was observed to be considerably smaller in the transgenic plants. The disease severity was evaluated by measuring the percentage of the infected sheaths and affected tillers at 7, 14 and 21 dpi (days post infection). The lesion on leaf sheath and the affected area on the tillers (43.78% and 39.22%, respectively) were minimum in the transgenic line as compared to those in the WT control (89.43% and 85.54%, respectively) at 21 dpi (Fig. 1a,b). However, in our earlier study, Jaldi-13 transgenic rice plant expressing *AtNPR1* had exhibited 50.84% ShB affected leaf area^20^. Similar results of *AtNPR1* and *BjNPR1* mediated enhancement of ShB resistance have been reported earlier^21,27^. ShB symptom development is a common phenomenon in the presence of suitable conditions; the disease is able to advance upward and move to the uppermost leaf sheaths and sometime panicle (Fig. 1c). The leaves adjoining to the infected sheaths become turn yellow and die^28^. The collapse of the rice plants caused by an excessive fungal infection was observed in the non-transformed WT plants after 30 dpi, while the transgenic plant lines overexpressing the *AtNPR1* gene restricted the vertical growth of the pathogen and facilitated to sustain the sheath and leaf architecture (Fig. 1d).

### Comparative leaf proteome between non-transformed wild-type and transgenic plants prior to and post *R. solani* infection

**Figure 2:**
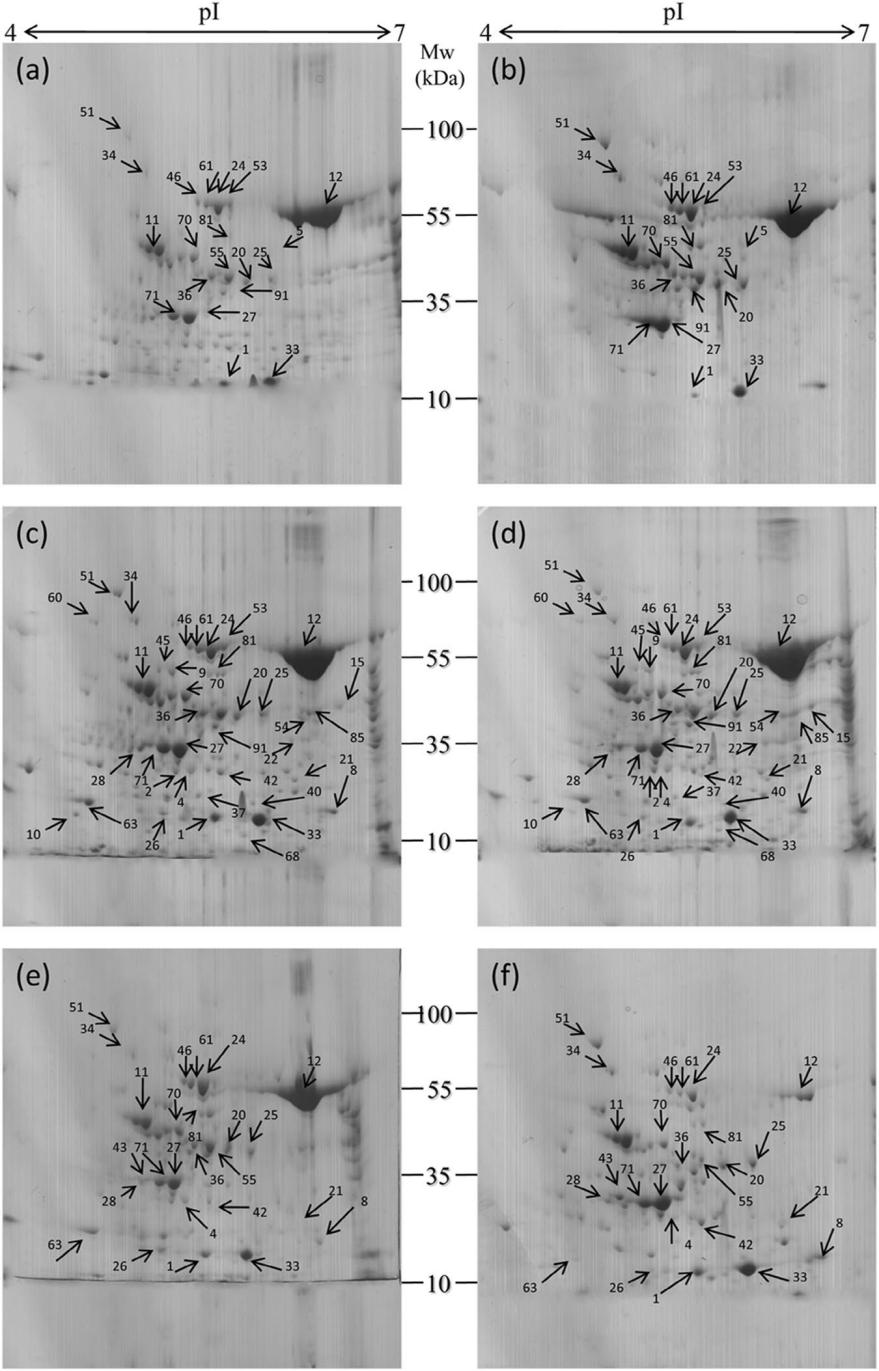
Representative 2-DE gel images of leaf proteins of WT and transgenic rice plants in three different conditions. (**a**) WT before infection, (**b**) transgenic before infection, (**c**) WT after 24 hr infection, (**d**) transgenic after 24 hr infection, (**e**) WT after 48 hr infection, (**f**) transgenic after 48 hr infection. 800 µg of total protein from each sample was loaded on IPG strips (17 cm), linear pH 4–7 gradient, and separated by 12% SDS PAGE.

To find out the effect of the transgene on leaf proteome, the protein profiles [before and after infection] of the non-transformed WT and the progeny of single homozygous transgenic line (105-5), which showed the highest level of ShB resistance among the tested *AtNPR1*-transgenic rice lines, were compared (Fig. 2). Similar to this present investigation, comparative proteome evaluation was also carried out with a single transgenic plant line in rice^29–31^, soybean^32^, and maize^33^. Although the leaf morphology was not largely affected post 24 and 48 h of infection, the molecular signalling network was speculated to be altered for generating a novel leaf proteome. In the present investigation, the identified protein spots were consistent in the all three replica gels (Supplementary Fig. S3). Image analysis of the 2-DE gels revealed an average of 222, 337, and 149 protein spots for WT at pre-infection, and post infection [one and two dpi (days post infection)], respectively, while 234, 345, and 136 protein spots were detected for transgenic plants at the same time points (Fig. 2). Out of 51, 110, and 98 spots that matched between the WT and transgenic plants, there were 20, 40, and 22 protein spots significantly modified for the three-time courses, respectively. Identified protein spots of >2.0-fold and <0.5-fold accumulation (significance level of *P* < 0.05), were considered DEPs (differentially expressed protein). Mean coefficient of variation of the spot’s intensity for the three experimental sets in non-transformed WT and transgenic plants prior to and post ShB infection was 40.32. Identified peptides were searched against the NCBI database using the MASCOT software and as a result16 up-regulated and 2 down-regulated peptides prior to infection, 21 up-regulated and 14 down- regulated peptides post 24 hpi (hours post infection), and 12 up-regulated and 10 down-regulated peptides post 48 hpi were identified in the transgenic leaves (Table 1). The mass spectra of the protein spots were searched using MASCOT against the NCBI database. The fold-change in the identified proteins in transgenic plants represented the ratio of change in spot intensity as compared to its non-transgenic WT counterpart (Table 1).

**Table 1:** Differentially expressed proteins identified using MALDI-TOF MS/MS in transgenic vs wild type rice plants before and after infection with sheath blight fungus *R.solani* [(>2.0-fold represents up-regulation and <0.5-fold represents down-regulation)].

Spot no.	Protein identified	Gi no.	Experimental Mol wt (kDa/pI)	Theoretical Mol wt (kDa/pI)	Protein score	Sequence coverage (%)	Relative fold change		
							BI	AI (24 hr)	AI (48 hr)
							Transgenic Vs WT	Transgenic Vs WT	Transgenic Vs WT
	**Cellular redox homeostasis**								
1	2-Cys peroxiredoxin BAS1	gi :4329578	24.307/5.37	28.307/5.67	260	37%	0.346	5.27	3.03
8	1-Cys peroxiredoxin A	gi:4344045	29.874/5.87	24.228/5.97	117	22%		7.95	4.02
	**Energy and Primary metabolism**								
15	Fructose-bisphosphate aldolase	gi:4344545	44.208/6.25	42.208/6.38	213	55%		5.22	
42	Proteasome subunit alpha type-3	gi:4327357	32.654/5.37	27.506/5.75	101	37%		0.28	3.12
2	Triosephosphate isomerase	gi:4347691	32.213/5.02	27.274/5.38	130	41%		4.76	
4	Cysteine synthase	gi:4334101	32.109/5.22	33.931/5.39	61	38%		2.32	0.2
12	Ribulose bisphosphate carboxylase large chain	gi: 4126887	54.956/6.24	53.418/6.22	120	20%	2.45	0.35	0.18
36	Glutamine synthetase	gi:4337272	47.987/4.89	46.956/5.96	107	30%	2.67	6.1	2.35
71	1, 2-dihydroxy-3-keto-5-methylthiopentene dioxygenase 4	gi:4348647	35.43/4.91	22.063/5.07	65	39%	5.45	0.46	5.13
54	Inositol-tetrakisphosphate 1-kinase 5	gi:4349492	40.976/6.17	38.106/6.04	89	19%		0.25	
9	ATP synthase subunit beta	gi:4339546	54.876/4.93	53.978/5.38	137	50%		6.76	
61	4-hydroxy-3-methylbut-2-en-1-yl diphosphate synthase	gi: 4329911	78.90/5.34	82.679/5.64	90	23%	3.98	5.21	0.38
63	Calmodulin-1	gi: 4324384	17.045/4.12	16.878/4.11	55	44%		3.46	0.43
68	Actin-depolymerizing factor 6	gi:4336623	16.453/5.63	16.045/5.55	63	41%		0.38	
11	FAD synthetase	gi:4334460	42.653/4.70	43.993/4.60	97	26%	3.89	6.23	4.21
	**Stress and defense**								
33	18.0 kDa class II heat shock protein	gi:4325342	18.03/5.60	18.133/5.61	143	27%	3.21	7.76	8.77
91	Heat stress transcription factor A-2A	gi:4334080	39.543/5.32	41.166/6.49	211	9%	3.26	5.56	
24	Endo glucanase 15	gi:4338100	69.456/5.41	68.963/8.30	132	17%	3.34	5.44	0.24
10	Salt stress root protein RS1	gi:4324284	18.453/4.25	21.788/4.92	100	34%		0.37	
40	Cyanate hydratase	gi:4348870	19.453/5.74	18.653/5.61	83	5%		0.23	
53	Beta-glucosidase-like SFR2	gi:4351127	75.876/5.45	73.624/6.28	123	21%	0.176	0.51	
20	Probable trehalose-phosphate phosphatase 2	gi:4349333	42.654/5.45	42.779/5.84	84	25%	3.02	4.74	
28	Probable protein phosphatase 2C1	gi:47525230	34.732/4.21	35.632/4.91	110	18%		4.26	5.1
55	Patatin-like protein 2	gi: 4345425	45.89/5.34	44.751/5.53	68	25%	2.76		0.25
70	Enolase	gi:4348176	48.234/5.23	48.285/5.41	80	22%	2.42	0.38	0.26
43	14-3-3GF14-f	gi:4333880	36.765/4.78	29.274/4.81	174	16%			20.31
	**Signal transduction**								
5	Plant intracellular Ras-group-related LRR protein 2	gi:4329845	53.231/5.53	55.345/5.51	79	20%	5.01		
21	Auxin-responsive protein IAA3	gi:4325890	28.022/5.95	28.264/6.48	120	29%		0.378	5.67
60	P-loop NTPase domain-containing protein LPA1	gi:4347923	80.023/4.38	79.076/7.56	172	17%		3.29	
25	Mitogen-activated protein kinase 6	gi:4349225	42.092/5.98	43.088/5.96	163	20%	5.87	6.67	8.01
	**Gene regulation**								
22	Protein arginine N-methyltransferase 7	gi:4339860	43.785/5.70	42.918/5.42	87	18%		3.19	
51	FACT complex subunit SPT16	gi:4335478	105.456/4.30	119.085/5.41	156	17%	8.78	0.34	6.94
	**Transport**								
45	Cation transporter HKT1	gi:4341971	55.343/4.32	59.542/8.43	80	20%		0.28	
	**Translation**								
26	Elongation factor Ts, mitochondrial	gi:4345504	43.098/6.43	40.217/8.54	70	22%		3.22	0.321
	**Others**								
46	Probable indole-3-acetic acid-amido synthetase, GH3.11	gi:4344247	60.002/5.10	69.889/5.32	84	17%	4.12	0.41	0.123
81	Delta-aminolevulinic acid dehydratase	gi:4341997	48.231/5.43	46.655/5.81	134	20%	3.33	2.12	0.278
85	Probable 4-hydroxy-tetrahydrodipicolinate reductase 1	gi:4329224	39.976/6.26	37.699/6.56	121	23%		5.01	
37	Probable calcium-binding protein CML12	gi: 4324100	26.7/5.47	18.574/4.83	89	17%		0.48	

### Identification of differentially accumulated proteins prior to and post sheath blight infection

**Figure 3:**
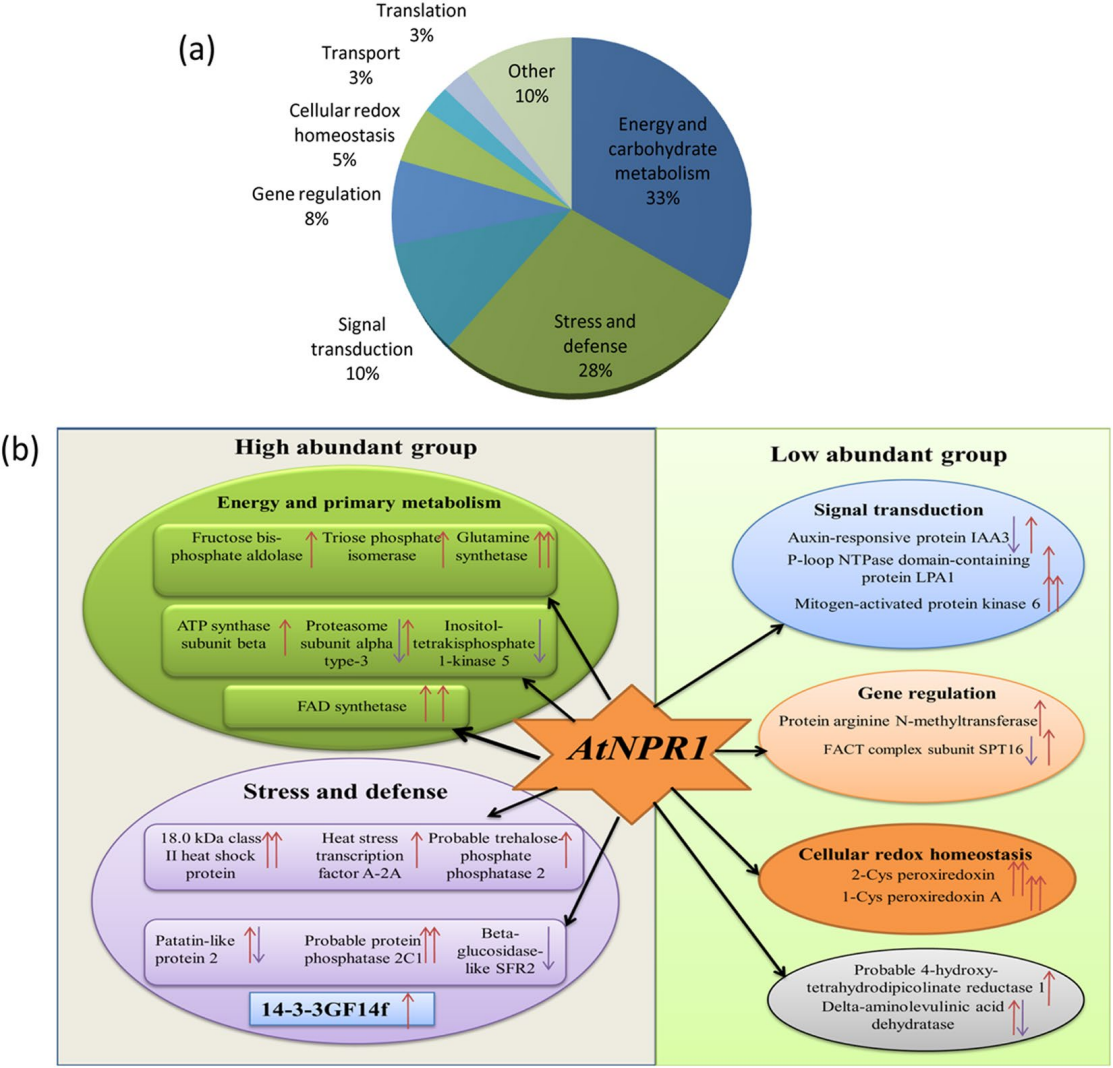
Functional classification of identified proteins and relative protein abundance. (**a**) Pie chart showing functional categorization of identified proteins based on biological process. (**b**) Network of different high and low abundant proteins downstream to *AtNPR1* found from 2D-proteomics data analysis after 24 and 48 hpi.

**Figure 4:**
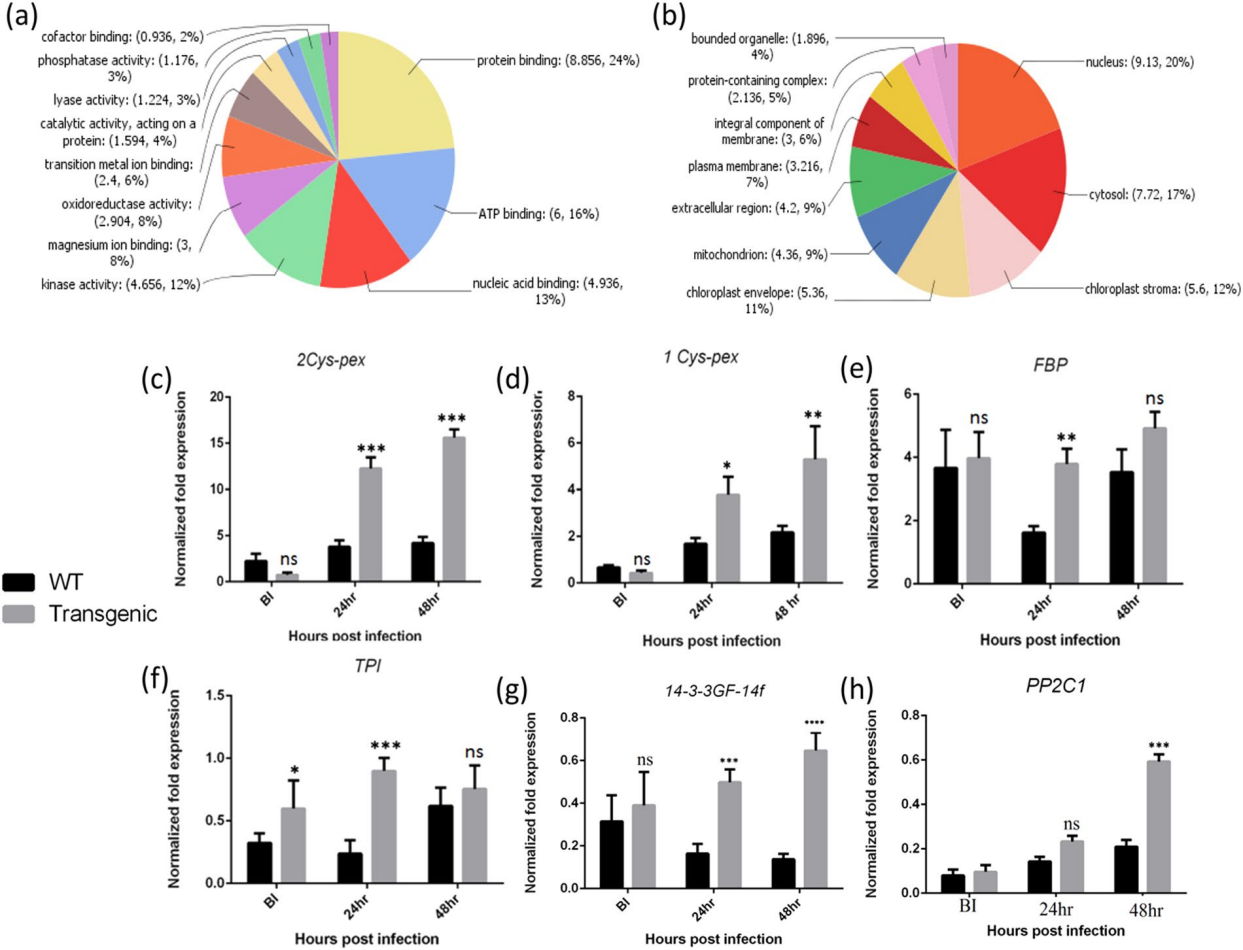
Classification of identified proteins based on molecular process and cellular component, and validation of protein expression by qRT PCR analysis. (**a**) Pie chart showing functional categorization of identified proteins based on molecular process. (**b**) Pie chart showing functional categorization of identified proteins based on cellular component. (**c**–**h**) The fold changes of six different genes resemble the differential accumulation of proteins although the degree of fold changes at transcriptional level is not same to their corresponding level of protein accumulation.

In order to find out the biological processes that were altered by the inoculation of the *AtNPR1*-transgenic rice plants with *R. solani*, KEGG allowed the classification of the peptides (DEPs) into eight functional groups on the basis of their biological process (Fig. 3a). The DEPs were associated with various metabolic pathways, such as the primary metabolism (33%), stress and defence (28%), cellular redox homeostasis (5%), signal transduction (10%), gene regulation (8%), translation and transport (3%), and the other categories (10%) (Fig. 3a). Therefore, the proteins associated with primary metabolism, stress and defence (highly abundant), and different signalling network (low abundance) were the major concerns in the present investigation (Fig. 3b). The categories of molecular function and cellular components identified 11 and 10 functional groups, respectively, in the proteins (Fig. 4a,b). The main functional categories of the significantly altered metabolic processes involved were glycolysis, lipid biosynthesis and alpha amino acid biosynthetic process. Identified proteins associated with organic hydroxyl compounds and responses to the toxic substrate were also observed to have markedly increased (Supplementary Fig. S6). Interestingly, the proteins associated with energy and primary metabolism, defence and defence signalling were observed to accumulate significantly in the transgenic leaves at 24 and 48 hpi compared to WT (Table 1). A previous report revealed that primary metabolism plays a significant role for supplying energy during the plant -pathogen interactions^34^. Another study revealed that energy is critical during plant defence responses for the expression of hundreds of genes from multiple defence-related pathways^35^. This is similar with the results of the present investigation. However, the other categories (10%) of differentially expressed proteins accumulated during stress. The identification of these novel proteins may dissect the possible role of *AtNPR1* protein in the transgenic rice plants for sheath blight resistance.

### Role of energy and primary metabolism related proteins pre and post sheath blight infection

The group belongs to energy and carbohydrate metabolism consisted with 13 differentially expressed proteins, among them fructose-bisphosphate aldolase, triose phosphate isomerase, glutamine synthetase, cysteine synthase, ATP synthase subunit beta, and FAD synthetase were observed to be up-accumulated in the transgenic plants post ShB infection. Five proteins that belonged to the energy and primary metabolism group were observed to be down-regulated at 24 hpi in the transgenic leaves, while 4 of the proteins from this category were significantly induced at 48 hpi compared to the WT leaves (Table 1). The biosynthetic alteration of key proteins belongs to the energy and primary metabolism groups in transgenic rice plants delineated increased rate of metabolism that has been previously discussed.

An earlier study demonstrated that overexpression of fructose bisphosphate aldolase in transgenic plants showed increased accumulation of hexose, sucrose, and starch with respect to wild-type plants^36^. An increase in the plastid aldolase activity is known to stimulate the loading of sucrose into the phloem and a swift transport of the synthesised sucrose to phloem instead of its accumulation in leaves^37^. TPI (triose phosphate isomerase) performs a vital role in glycolysis and is responsible for energy production. An earlier report demonstrated that in transgenic potato roots, a huge decrease in the cytosolic triosephosphate isomerase hampers the carbon distribution in primary metabolism^38^. Therefore, the upregulation of these proteins post ShB infection (24 hpi) in the transgenic leaves reflects an increase in the carbohydrate biosynthesis. *Gln-α* (Glutamine synthetase) obtained from *Proteus vulgaris* was inducible to pathogenic as well as non-pathogenic strains of fungi, signifies that it acts as an early-responsive defence gene^39^. In addition, the regulation of cytosolic Cys homeostasis has been demonstrated as a critical factor for orchestrating the plant response to pathogens^40^. The upregulation of glutamine synthetase and cysteine synthase in the transgenic plants under pathogen stress enhances the rate of amino acid biosynthesis, reflecting an increased stress tolerance in comparison to that in the non-transformed WT plants. The expression of *Arabidopsis NPR1* (*AtNPR1*) in transgenic rice plants facilitates the increment of carbohydrate and amino acid biosynthesis, which has also been evidenced from the upregulation of fructose bisphosphate aldolase, triose phosphate isomerase, glutamine synthetase, and cysteine synthase post *R.solani* infection (Table 1). This result revealed that the transgenic rice plants maintain a stable rate of carbohydrate and amino acid biosynthesis with respect to non-transformed WT plants during *R solani* infection.

Adenosine triphosphate (ATP) synthase is involved in ATP synthesis/hydrolysis and subsequently transport other substances through the membrane. In the present investigation, an ATP synthase beta subunit was upregulated post infection (24 hpi). ATP synthase plays a critical role in the host-pathogen interactions, and also linked to oxidative phosphorylation and ATP synthesis^41^. This finding supported the fact that the transgenic plants exhibit a higher demand for energy during the ShB infection.

A previous study demonstrated that a large number of proteins involved in photosynthesis are altered during the plant-pathogen interactions, implying that the pathogen has a strong influence on the host photosynthetic machinery^42^. Similarly, in the present study, a significant down-regulation of large chain of RuBisCO (Ribulose bisphosphate carboxylase) was observed in the transgenic rice leaves as compared to the non-transformed WT at both 24 hpi and 48 hpi (Table 1). The down-regulation of certain photosynthetic proteins during host-pathogen interaction may relies on certain feedback control of the photosynthetic genes^43^. The 20S proteasome, a proteolytic core of the 26 proteasomes, is involved in the degradation of oxidised and defective proteins^44^. In the present study, the elevation in the levels of the proteasome subunit alpha type–3 observed at 48 hpi in the *AtNPR1* transgenic line might play a protective role against oxidative stress through the degradation of defective proteins by the other oxidising enzymes, and it might be able to cope better against the patho-stress.

The actin cytoskeleton has been known to be involved in plants defence response against fungal pathogen^45^. The ADF (Actin-depolymerizing factor) has been reported to negatively modulate the wheat resistance against *Puccinia striiformis*^46^. The results of the present investigation also demonstrated a down-regulation of the ADF6 protein in *AtNPR1* transgenic rice plants. Therefore, overexpression of *AtNPR1* in the transgenic rice line provides better defence against the stress through the maintenance of a stable architecture of actin cytoskeleton in comparison to the non-transformed WT plants.

### Role of stress and defence related proteins during sheath blight pathostress

Stress and defence related proteins were second most abundant group under the biological process, consisted of 28% of total proteins. HSPs are known to play a vital role after infections, thereby preventing the damage to host plant^47^. The present study revealed an up-regulation of a 18.0-kDa class II Hsp and a heat-stress transcription factor A–2A post infection (24 and 48 hpi) in the transgenic *AtNPR1*-overexpressing plants in comparison to non-transformed WT (Table 1). Based on previous observations^48^ and with the outcome of the present investigation, it is hypothesized that transgenic rice plants overexpressing *AtNPR1*, compared to the WT plants, could participate in the primary stages of defence response against *R.solani* via the elevation of different groups of HSPs.

Another previous report confirmed that the overexpression of *OsBIPP2C2a* in transgenic tobacco plants resulted in elevated disease resistance and the activation of several defence-related genes against the blast fungus *Magnaporthe grisea*^49^. This result was in congruence with the present study, where a probable protein phosphatase 2C1 was significantly upregulated post-ShB infection at 24 and 48 hpi (Table 1). Therefore, the overexpression of the *AtNPR1* gene also upholds the levels of protein phosphatase 2C1 in the leaves, thereby exhibiting stress tolerance in the transgenic rice plants presumably by the activation of the defence related gene.

Another enzyme related to biotic stress responses—probable trehalose-phosphate phosphatase 2—was observed to be elevated in the transgenic plants as compared to the WT plants at 24 hpi (Table 1). The enzyme named as probable trehalose-phosphate phosphatase 2 catalyses the production of free trehalose. A previous study reported that application of trehalose in wheat plants provided low level of protection against the powdery mildew fungus *Blumeria graminis*^50,51^. The upregulation of trehalose-phosphate phosphatase 2 post sheath blight infection resembled free trehalose-mediated stress response in the transgenic plants.

In the present study, Patatin-like protein–2 was observed to be downregulated in the leaves of transgenic plants at 48 hpi. This protein was effectively identified in several plants and has been involved in host plant defence though Jasmonic acid or oxylipin signalling^52^. Indeed, PLP2 (Patatin-like protein–2) expression favoured the development of necrotising pathogens and increased the resistance to obligate pathogens^53^. The down-regulation of patatin-like protein–2 post sheath blight infection in the transgenic leaves was believed to inhibit fungal colonisation and demonstrated an SA-mediated stress response in plants.

A novel protein 14–3–3GF14f was observed to be present in high amount, showed 20.31-fold up-accumulation in transgenic rice plants at 48 hpi (Table 1). But the characterization of this important protein remained to be performed. Present results indicate that the overexpression of the *AtNPR1*in the transgenic rice plants during the sheath blight infection stimulates the expression of 14–3–3GF14f gene. Therefore, the 14–3–3GF14f protein could act as a potential candidate protein for the deployment of sheath blight-resistant rice plants in the future.

### Differential accumulation of signalling related proteins

A protein related to signalling and gene regulation, referred to as mitogen-activated protein kinase 6 (MAPK6), was identified and observed to be specifically accumulated in the transgenic material at one and two dpi (days post infection) (Table 1). The transduction of appropriate defence signal is vital for the survival and adaptation of different plants to stress conditions. For instance, the MAPK mediated stress signalling performs an important step for the setting up of defence response against pathogens^54^. A recent study demonstrated that the MAPK6 modulates the expression of *NPR1* gene and promotes the leaf senescence induced by SA^55^. Similarly, the auxin-responsive protein IAA3 exhibited variable expression at 24 and 48 hpi in the present study (Table 1). The interaction of auxin with the other hormones for mediating the defence responses has previously been reported. SA, a phytohormone participated in the defence responses in plants, has been reported to repress the auxin signalling pathway as a part of the disease resistance mechanism^56^. Therefore, the overexpression of *AtNPR1* in the transgenic plants activated *PR* and defence-related genes by significantly modulating the MAPK6 and IAA3 signal transduction pathways.

### Differential accumulation of ROS homeostasis-related proteins

In the present study, the increased abundance of 2-cys peroxiredoxin and 1-cys peroxiredoxin A post-ShB infection might have lead to provide resistance at the initial phases of fungal infection (Table 1). The quantitative Real Time PCR (qRT-PCR) analysis also revealed an elevation in the mRNA abundance of the two peroxiredoxin genes, displaying similar to the changes in the protein expression (Fig. 4c,d). The higher abundance of 2-cys peroxiredoxin and 1-cysperoxiredoxin A in the leaves of the transgenic plants indicated increased ROS homeostasis and tolerance against oxidative stress post the sheath blight patho-stress as compared to that in the WT plants.

### Accumulation of other proteins during sheath blight infection

Along with the physiological alteration during the sheath blight infection, other proteins involved in secondary metabolism have also been implemented in the present study. Certain proteins such as probable delta-aminolevulinic acid dehydratase were observed to be induced in the leaves of the transgenic rice plants, while probable indole-3-acetic acid-amido synthetase GH3.11 was down-regulated (Table 1). Probable 4-hydroxy-tetrahydrodipicolinate reductase 1 was seemed to be increased in the transgenic rice plants with respect to its level in WT, which assisted in increasing the lysine biosynthetic process during the patho-stress. Delta-aminolevulinic acid dehydratase was elevated in the transgenic leaves prior to and post-infection in comparison to the WT plants, thereby assisting in increasing the tetrapyrrole synthesis. These tetrapyrroles perform critical functions in important biological processes i.e. the detoxification of ROS (Reactive oxygen species), the assimilation of nitrate and sulphate, respiration, PCD (programmed cell death) and the light-harvesting reactions of photosynthesis^57^, thereby providing better resistance to sheath blight patho-stress in the transgenic rice plants as compared to the non-transformed WT plants.

### Validation of proteomic data through gene expression analysis

Proteins involved in primary metabolism, stress and defence, and ROS homeostasis were chosen for expression analysis in all three time points i.e. pre and post sheath blight infection. The differential expression of the transcripts of two ROS homeostasis related genes, i.e. 2-cys peroxiredoxin and 1-cys peroxiredoxin A in the transgenic rice plants resembled the variable accumulation of their consecutive proteins (Fig. 4c,d). Fructose-bisphosphate aldolase (FBP) and triose phosphate isomerase (TPI) accumulated in the transgenic plants in 24 hpi exhibited 2.18-fold and 3.07-fold upregulation at the transcript level (Fig. 4e,f). The highest upregulation [3.2-fold and 4.33-fold] for the 14–3–3GF14f transcript in the leaves of transgenic plants was obtained at 24 and 48 hpi, which paralleled with the proteomics data (Fig. 4g). The transcript levels of protein phosphatase 2C1 were demonstrated to be induced in the transgenic leaves by 3.04-fold only at 48 hpi, which was also similar to the proteomics data (Fig. 4h). But, there is no definite correlation between the levels of gene expression with their corresponding levels of protein.

### Protein-protein interactions among differentially accumulated proteins

Sheath blight infection induces a complicated signalling network that involves interactions among numerous proteins and metabolites with diverse functions. The signalling network of highly abundant differentially expressed proteins that belongs to primary metabolism, biotic stress and defence, and other low-abundance proteins downstream to *AtNPR1* in the WT and transgenic plants is depicted (Fig. 3b). The interactions among 38 differentially accumulated proteins forms two definite clusters, which showed interaction between themselves (Supplementary Fig. S4). The interaction of proteins belongs to stress and defence with the proteins related to energy and primary metabolism revealed a complex signalling crosstalk that activates the expression of downstream defence related genes in the transgenic rice plants. Under sheath blight patho-stress, energy requirement is considerably much higher in order to synthesise proteins related to stress and defence along with the certain proteins belongs to metabolism. The experiments in the present study revealed that the biosynthesis of carbohydrates was subdued under the sheath blight infection, followed by low level of energy production in the non transformed WT plants, and the plants challenged for energy. The increment of sugar metabolism in the transgenic rice plants under ShB infection also supported this result (Supplementary Fig. S5). On the contrary, transgenic sheath blight resistant rice plants could regulate their metabolism in such a way that the increase in sugar production supplied the extra energy required for the production of more proteins related to stress and defence. In addition, the elevation in the stress and defence-related as well as genes related to defence signalling pthways is a essential regulatory phenomenon in plants for combating myriad stress responses in addition to sheath blight infection.

### Comparative analysis of differential metabolites in the transgenic and non-transformed wild-type rice plants pre and post *R. solani* infection

Subsequent to the detection of differential proteome response in the transgenic rice pre and post *R.solani* infection, the variation in the metabolite pools challenged with *R. solani* were further explored. Using GC-MS, a number of compounds were observed to interact with different enzymatic pathways also precipitated in the rice-sheath blight interaction in the proteomic analysis. A total of 40 common metabolites were detected in the non-transformed WT and transgenic leaf samples. Out of these, 7, 22, and 23 metabolites were induced in the transgenic samples at three time points [Pre and post (24 and 48 h) infection], whereas 1 and 6 metabolites were down-regulated at 24 and 48 hpi (Table 2). The host respiratory machinery, including glycolysis and TCA cycle, was shown to be elevated in the transgenic rice plant post the *R. solani* infections compared to WT, as apparent in both proteome and metabolome data. A previous study revealed that plants subsequently increase their respiration rate in order to gain energy for producing and mobilising the stress and defense realted compounds to meet the metabolic needs developed following pathogen attack^42^. Several resistance-related metabolites, such as myristic acid, silanol, myo-inositol, arabitol, malic acid, ribonic acid, sucrose, erythrose and fructose, were significantly up-accumulated in the transgenic plants post ShB infection (24 and 48 hpi) as compared to non-transformed WT. The metabolite L-arabitol was detected at a retention time of 19.138 min. Arabitol concentration was observed to be elevated dramatically as the infection progressed in the transgenic plants (2.87-fold and 2.47-fold elevation at 24 and 48 hpi). In a previous study, D-arabitol was demonstrated to act as a quencher of reactive oxygen species in host defence^58^. Sugars participate in several metabolism and downstream signalling network in plants^59^. Sugars mainly fructose, erythrose and sucrose (2.52, 3.32- and 3.13-fold at 48 hpi) that play a typical role as the carbon and energy sources are recognised as signalling entity in plants^59^. Sugar signals were involved in activating immune reactivity against different pathogens and it is possible that they induce PAMP (Pathogen Associated Molecular Pattern) triggered and effector-triggered immunity (ETI) in plants. MI (Myo-inositol) is a precursor for numerous compounds, including the ones that possess possible antioxidant activity^60^. MI may also be involved in ascorbate synthesis^61^. The up-accumulation of myo-inositol in transgenic plants post infection might promote ROS homeostasis in the transgenic tissues thereby preventing oxidative damage as compared to non-transformed WT plants.

**Table 2:** Metabolic alternation in transgenic vs wild type rice plants before and after infection with sheath blight fungus *R. solani* (>1.5 represents up-regulation and <0.6 represents down-regulation).

Metabolite	Category	RT(Retention time) (Min)	Before infection (Mean ratio transgenic vs WT)	After infection (24 hpi) (Mean ratio transgenic vs WT)	After infection (48 hpi)(Mean ratio transgenic vs WT)	Function/Pathway
D-Fructose	Sugar	33.46	0.79	1.16	2.52	Glycolysis
D-Erythrose		19.48	1.25	0.68	3.32	Isomer of sucrose
D-Talose		22.09	1.30	1.39	2.22	Aldohexose sugar
Sucrose		46.56	1.39	1.42	3.13	Sugar transportation
D-Turanose		31.39	1.55	3.92	1.74	Isomer of sucrose
D-Tagatofuranose		19.92	1.33	0.53	0.52	Unknown
Arabino-Hexos-2-ulose		10.363	1.02	1.37	0.66	Role in stress tolerance
Oxalic acid	Organic acids	8.33	1.21	1.16	1.60	Virulence determinant
Galactaric acid		23.41	1.10	4.22	1.83	Unknown
Propanoic Acid		6.81	2.88	1.03	1.39	Role in plant growth
Glycolic acid		7.15	0.97	1.49	0.41	Photorespiratory pathway
Butanedioic acid		12.03	1.29	1.22	1.51	Growth promoting compound
Malic acid		15.290	1.18	4.99	3.15	Role in plant defense
Ribonic acid		23.442	1.06	2.83	1.27	*Role in stress response*
Quininic acid		21.26	2.38	2.76	0.29	Quinate metabolic pathway
Benzoic Acid		15.630	1.37	1.14	1.01	Biosynthesis of secondary metabolites
Glyceric acid		12.32	3.64	1.74	2.28	Oxidized product of glycerol
Shikimic Acid		20.57	1.36	2.14	0.10	Biosynthesis of aromatic amino acid
L-Serine	Amino acids	10.858	0.93	2.61	1.19	Biosynthesis of phospholipids & sphingolipids
L-Aspartic acid		14.12	1.15	1.42	1.66	Pyrimidine metabolism
L-Glutamic acid		17.56	1.49	3.25	1.39	Signalling molecule in plant defense
L-Threonine		11.62	0.89	2.24	2.56	Confers resistance against pathogen
Pyroglutamic acid		15.53	1.00	1.88	1.89	Role in plant growth
L-Arabitol	Sugar alcohols	19.138	1.31	2.87	2.47	Unknown
Silanol		11.22	1.51	1.09	2.42	Role in plant defense
Myo-Inositol		24.52	1.10	2.09	2.72	Decrease oxidative stress
Phytol	Fatty acids	25.65	1.06	2.12	1.38	Chlorophyll breakdown product
Stearic acid		26.54	1.29	2.25	2.55	Fatty acid biosynthesis
L-Threonic acid		16.23	0.88	2.45	0.58	Ascorbic acid metabolism
Myristic Acid		18.47	1.12	2.56	3.87	Fractionated saturated fatty *acid*
Palmitic Acid		23.955	1.21	1.34	1.49	Structural role in plant cell membrane
Pentenoic acid		24.034	3.15	1.85	2.15	Unknown
Linoelaidic acid		26.106	1.75	2.72	1.21	Isomer of linoleic acid
9, 12-Octadecadienoic Acid		26.185	1.02	1.29	1.72	Role in plant defense
Phosphoric Acid	Others	9.23	1.30	3.45	0.55	Source of phosphate
Neophytadiene		21.68	1.28	1.84	2.46	A class of sesquiterpenoid
2-Hexadecene		21.13	0.99	1.28	1.19	Unknown
3, 7-Dioxa-2, 8-Disilanonane		22.293	1.13	1.40	0.90	Unknown
Campesterol		41.991	0.91	1.44	2.01	Role in growth and development
Stigmasterol		42.74	1.16	2.69	2.01	Role in plant pathogen interaction

Silanol is related to the hydroxy functional group, and able to alleviate plant disease either through the prevention of pathogen penetration through structural reinforcement^62^ or through an increase in plant resistance via activation of multiple defense signalling pathways^63^. The up-regulation of silanol in transgenic rice plants as compared to non-transformed WT at 48 hpi resembled activation of defence gene and signalling pathway which is seemed to play a vital role in the sheath blight patho-stress. In the present investigation, the levels of malic acid reached 4.99-fold and 3.15-fold at 24 and 48 hpi in the transgenic rice plants, respectively, which suggested that the *AtNPR1* gene upholds the levels of TCA cycle and higher energy production for defence gene activation in the transgenic plant material. An elevated level of NADP-ME may serve as structural framework and energy production for synthesis of defence compounds, depicting a possible role of malate biosynthesis and metabolism in plant defence^64^.

Marked accumulations of aspartic acid, glutamic acid, threonine, and serine were observed in the transgenic *AtNPR1*-overexpressing plants post infection with respect to non-transformed WT; all of these compounds are known to accumulate in plants under stresses, and converted to resistant metabolites in the plants in order to circumvent these stresses^65^.

The accumulation (2.14-fold) of shikimic acid in transgenic plants at 24 hpi suggested higher aromatic amino acids production and induction of phenylpropanoids metabolism. The well-known regulatory roles of phenylpropanoids include biosynthesis of salicylic acid (SA), a master regulator of systemic acquired resistance, quencher of reactive oxygen species, and programmed cell death^66^.

### Expression analysis of various endogenous defense-related genes prior to and post *R. solani* infection

**Figure 5:**
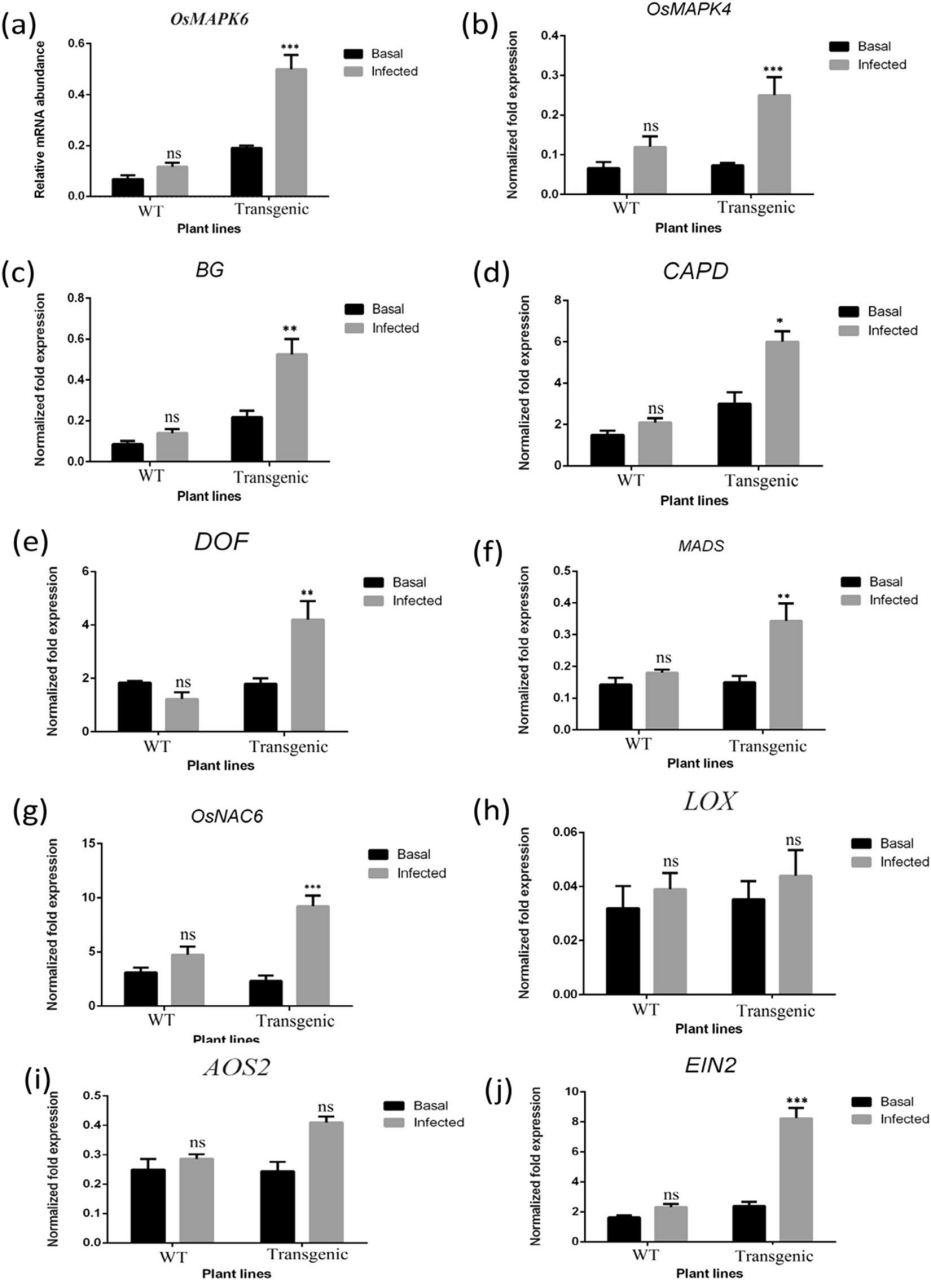
Real-time PCR analysis of some differentially expressed Stress marker, transcription factors, programme cell death, and JA pathway genes in transgenic and WT rice plants (Basal and 24 hr post infected condition). Expression of (**a**) *OsMAPK6*, (**b**) *OsMAPK4*, (**c**) *BG*, (endo-1, 3-1, 4-ẞ-glucanase mRNA of *Oryza sativa*) (**d**) *CAPD*, (polysaccharide biosynthesis protein) (**e**) *DOF*, (Dof zinc finger) (**f**) *MADS*, (**g**) *OsNAC6*, (**h**) *LOX*, (**i**) *AOS2*, and (**j**) *EIN2* gene in transgenic and wild type plants. Each bar represents the mean ± SE of three independent experiments. Experiment performed by SYBR green-based quantitative real-time PCR, using *β-tubulin* as internal control.

In order to identify the possible effect of sheath blight infection on rice plants, the expression analysis of several marker genes selected from the non-transformed WT and transgenic plants prior to and post-infection (24 hpi) were analysed using qRT-PCR (Fig. 5). Fungal structural component MAMPs (microbe-associated molecular patterns), such as chitin oligomer, had been known to activate *OsMAPK6*, which in turn plays a vital role in the biosynthesis of phytoalexins such as momilactone and phytocassane^67^. In the present study, we obtained a significant upregulation of the *OsMAPK6* gene post infection in the transgenic rice plants as compared to non-transformed WT (Fig. 5a). The *OsMAPK4* gene is known to be induced by exogenous application of Jasmonic Acid (JA), infection with *Magnoporthe grisea* as well as death of host cell^68^. In the present study, *OsMAPK4* was observed to be induced by more than 2.04 folds post infection in the transgenic rice leaves at 24 hpi (Fig. 5b). The expression levels of *ẞ-1*, *3-glucanases*, which are the markers for plant defence responses, exhibited 2.33-fold induction in the transgenic rice lines post ShB infection as compared to non-transformed WT (Fig. 5c). A probable role for *ẞ-1*, *3-glucanases* in the structural defence of plants against pathogenic microorganism has been reported because ẞ-1, 3-glucan is a major cell wall components of several pathogenic as well as non pathogenic fungi^69^. Furthermore, the release of oligomers and monomers from the cell walls of pathogenic fungi serves as a signal for the elicitation of host defence responses^70^. Callose, an important carbohydrate compound, plays a significant role in plant defence response by blocking the penetration of fungus into the plant cells^71^. Similarly, genes responsible for callose synthesis (CAPD) were observed to be upregulated in the infected transgenic rice plants with respect to non-transformed WT (Fig. 5d). Dof zinc fingers, which have been reported to be upregulated at the time of pathogen attack and other defence- stimulus in previous studies^72^, were shown to be induced in the transgenic rice lines post ShB infection in the present study as well (Fig. 5e). Interestingly, the genes upregulated in the infected transgenic leaves also included MADS box and NAC6 transcription factor (Fig. 5f,g); these two well known genes have previously been reported to participate in stress management in different plants^73^. Disease resistance is a innate immune response of plants to activate several signal transduction pathways^74^. No distinguishable differences were observed in the expression profiles of the genes related to JA signalling pathways, namely, *LOX* and *AOS2* genes (Fig. 5h,i). A 3.43-fold up-accumulation of EIN2 transcript was observed post infection in the overexpressing transgenic line compared to WT (Fig. 5j). A previous study delineated that phytohormones such as ethylene (ET) performs a key role in stress signalling in response to microbial infection^75^.

## Conclusion

**Figure 6:**
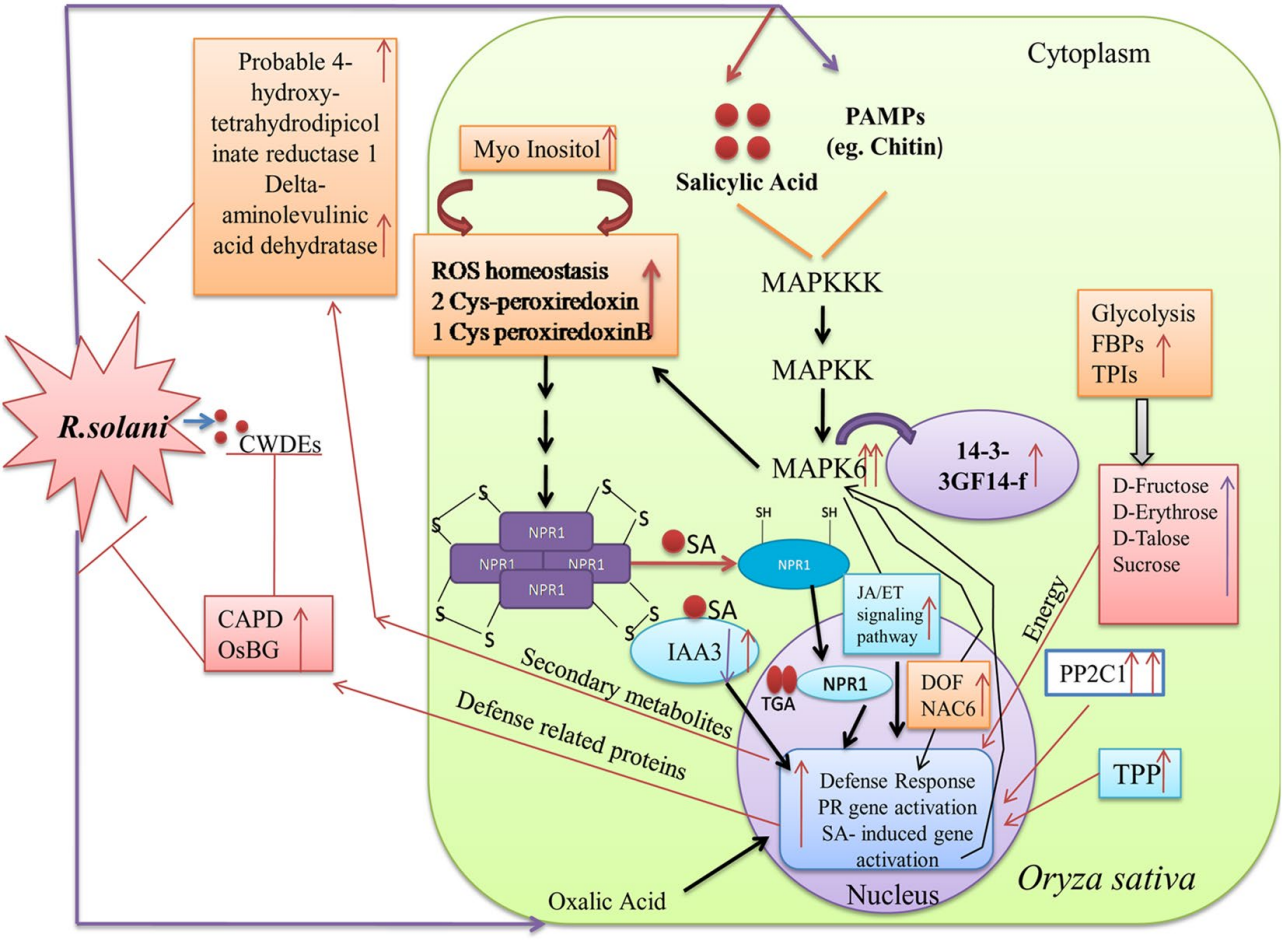
A hypothetical model summarizing alternation of metabolic and cellular pathways due to sheath blight patho-stress in *AtNPR1* overexpressing transgenic rice plants. Red arrow represents up-regulation, while violet indicates down-regulation. Double arrow indicates expression of protein in two different conditions (24 and 48 hpi). NPR1: Non expresser of pathogenesis related gene 1; CWDE: Cell wall degrading enzymes; SA: Salicylic acid; MAPK6: Mitogen activated protein kinase 6; PP2C1: Protein phosphatase 2C1; TPP: Trehalose protein phosphatase; FBPs: Fructose bis phosphatase; TPIs: Triose phosphate isomerase; IAA3: Auxin responsive protein; PAMPs: Pathogen associated molecular pattern. CAPD (polysaccharide biosynthesis protein); BG (endo-1, 3-1, 4-b-glucanase mRNA of *Oryza sativa*).

The present study opens up a novel dimension of a cross-talk between disease resistance and proteometabolomics, underlining the roles of signal transduction pathways and their associated proteins and metabolites in enhancing the resistance against ShB disease in the *AtNPR1-*transgenic rice. The present study involved an inclusive analysis in the proteomic alternations as well as the metabolic profile due to *R. solani* infection in the non-transformed WT and transgenic rice plant lines. Taken together, it was demonstrated that the stress-tolerance ability of *AtNPRI* could be attributed to a plethora of functionally divergent downstream proteins and metabolites including the defence, redox homeostasis, signalling, gene regulation, and cell metabolism ones (Fig. 6). Such a comprehensive study on novel proteins and metabolites harbours the potential to dissect the tolerance ability of transgenic rice plants and identify suitable candidate genes for providing pathogen resistance in the near future.

## Methods

### Plant material, growth parameters and pathogen inoculation assay

Rice (*Oryza sativa* L. Subspecies *indica*) cv. IR–64 and *AtNPR1*-overexpressing transgenic IR–64 line (105-5) (Supplementary Figs S1 and S2) were taken as the non-transformed WT sheath blight-suceptible and sheath blight-resistant rice lines, respectively. T_6_ homozygous progeny plants of 105-5 rice line were grown in controlled condition at 30/25 °C temperature and relative humidity of 70–80% in paddy soil enriched with fertilizer (N: P: K = 80:40:40 kg/ha). At maximum tillering condition, two sets of three plants (Three plants from wild type and three progeny plants from homozygous transgenic 105-5 rice line) were selected for inoculation. *Rhizoctonia solani* AGI-IA (Hyderabad isolate) was used for artificial inoculation for the sheath blight disease. The inoculum of the sheath blight fungus *Rhizoctonia solani* AG1–1A was prepared as described in a previous study^76^. Equal amounts of inoculum were placed amidst the tillers in each plant during highest tillerting condition. High humidity was maintained artificially in order to ensure high disease pressure. The physiology of the plants were observed and leaf samples were taken in three replicas from three consequtive time points—prior to and post sheath blight infection (24 h and 48 h) conditions—for proteomic study, metabolomic study, and qRT-PCR analysis. The leaf samples were stored in RNA later solution in order to isolate RNA which was then preserved at −80 °C.

### Protein extraction

Total protein from non-transformed WT and transgenic plants was extracted by following a previously described procedure^77^ with a few modifications. Briefly, the infected and non-infected leaf samples from both transgenic and wild-type plants (5.0 g) were crushed into fine powder immediately with liquid nitrogen and homogenised in 20 mL protein extraction buffer [20 mM Tris-base (pH 7.8), 1 mM PMSF, 10% (w/v) PVP, 1% (w/v) DTT and 1% (v/v) Triton X–100]. The extract was centrifuged at 13,000 rpm for 20 min at 4 °C. The upper aqueous phase was taken out and the phenol phase was extracted two times with same volumes of cold extraction buffer. Each extracted aliquots were finally mixed and precipitation of total protein was performed by mixing with it 100 mM ammonium acetate dissolved in cold methanol. The solution obtained was stored overnight at −20 °C. The centrifugation of aforementioned solution was performed at 8000 × g for 20 min. The pellet was thoroughly washed three times with ice cold acetone, and finally dissolved in lysis buffer [9M urea, 2M thiourea, 2% (w/v) CHAPS, 1% (w/v) DTT and 1% (v/v) carrier ampholytes of pH 3–10 (Bio-Rad, USA)]. The concentration of total protein was measured according to an earlier described procedure^78^ with BSA used as a standard solution.

### Two-dimensional polyacrylamide gel electrophoresis (2-DE)

The one-dimensional separation of total protein was performed using isoelectric focusing (IEF) according to a procedure mentioned in Bio-Rad manual book. 800 µg of total protein was taken for rehydration on 17-cm strips (Bio-Rad, Hercules, CA, USA) in a linear pH gradient (4–7). Isoelectric focussing (IEF) was conducted using the IEF Cell (Bio-Rad) with a procedure mentioned in Bio-Rad manual. The temperature of instrument during IEF was 20 °C and 50 mA electrical current was maintained per strip. After focussing, strips were equilibrated two times with equilibration buffer I and equilibrium buffer II, respectively, each for 15 min duration. Second dimension electrophoresis was performed with equilibrated strips in SDS-PAGE in a vertical slab of 12% acrylamide. PROTEAN Tetra Cell (Bio-Rad) instrument was used for these perpose. The protein gels were stained using the colloidal Coomassie Brilliant Blue R-350 and spots were visualised using a Calibrated Imaging Densitometer (Bio-Rad, GS–800). Three independent biological replicates were prepared for each of six sets of samples [WT and progeny transgenic plants prior to and post 24 and 48 h sheath blight infection].

### Image and data analysis

Subsequent to staining, picture of the gel was captured using the instrument Calibrated Imaging Densitometer (Bio-Rad, GS–800) and the picture was thoroughly analysed with the help of PD Quest Software version 8.0 (Bio-Rad, USA). All the protein spots in the gel were compared to the respective spots in the reference gel, and normalisation of each spot density was performed against total densities of gel. The percentage volume of each spot was calculated considering the value of three biological replicates. Statistical analysis (*t*-test) was performed in order to evaluate the significant differences between WT and transgenic plants respectively. Only those protein spots showing reproducible fold change (>2.0-fold for up-regulation and ˂0.5-fold for down-regulation) and significant differences (P < 0.05) were considered as DEPs (differentially expressed proteins).

### Trypsin digestion of protein spots and MALDI-TOF MS/MS analysis

The spots were excised manually from the gel and followed by trypsin digestion using a trypsin digestion kit (Pierce, USA). The trypsin digested samples were lyophilised and 5 µL of 0.1% TFA and 50% acetonitrile solution was used to dissolve the samples. A volume of 1 µL of the sample was taken for the identification of respective peptide using the MALDI-TOF-MS/MS analyser (Bruker Daltonics, Germany) and the peptide identification was performed using the MALDI-TOF-MS/MS analyser (Bruker Daltonics, Germany). Spectra of the peptides were collected using Flex Control software and Flex Analysis 3.4 software was used for data analysis. The respective proteins were searched by MASCOT program (Matrix Science, London, England) and identification was performed using the NCBI-nr protein sequence database (NCBI, Bethesda, MD, USA) which used MOWSE algorithm.

The data were screened against the NCBI-nr database with the help of the following parameters: taxonomy—*Oryza sativa* (25805290 sequences); cleavage specificity—trypsin with one missed cleavages allowed; allowed modifications—carbamidomethyl (fixed), oxidation of methionine (variable); cleavage by trypsin—cuts C-terminus side of KR unless the next residue is P. Only the significant hits (*P* < 0.05) were taken on the basis of MASCOT probability analysis.

### Isolation of RNA and qRT-PCR analysis

In order to validate the expression profile of certain well-known defence genes and certain genes corresponding to the selected protein spots, qRT-PCR was conducted. Extraction of total RNA was performed from leaves of individual rice plants (prior to and post infection) of the six sets with the help of the plant-RNA mini kit (Qiagen, USA) in accordance with the manufacture’s instruction. Three replicas were considered form each set. NanoDrop spectrophotometer (Smartspec, Bio-Rad) was used to measure RNA concentration. Subsequent to the removal of traces of DNA through DNase I treatment, 2 µg of total RNA was taken to synthesise cDNA with the help of the Maxima First Strand cDNA Synthesis Kit (Fermentas). 1 µL of cDNA was used as the template for each RT-PCR reaction. Primer 3 software was used to design respective primer sets. The qRT-PCR reaction was conducted using the transcript-specific primers (Supplementary Tables S1 and S2) and SYBR Green dye (Fermentas, Canada); the cycles were as follows: 95 °C for 30 s, 60 °C for 30 s and 72 °C for 30 s. The procedure used was in accordance with the manufacturer’s instructions (CFX 96 Real-time system, Bio-Rad). The quantitative variations between the different samples were evaluated using the 2^−^^ΔΔCT^ method^79^, and the *ẞ-tubulin* gene was used as an internal control to normalise all the data. In order to proper validation of the results, each experiment was conducted in triplicate.

### Total soluble sugar estimation

The estimation of total soluble sugar was performed spectrophotometrically using a previously described method^80^. The leaves of non transformed WT and transgenic rice plants, prior to and post (24 hpi and 48 hpi) ShB infection were homogenised using 80% ethanol and centrifuged at 3000 rpm for 10 min. The supernatant (1 mL) was taken out and mixed with 0.05% phenol (pH 6.0) and 2 mL of 98% sulphuric acid and incubation at 30 °C for 20 min. The OD (optical densities) of the solution was measured using a spectrophotometer (Smartspec, Bio-Rad, USA) at 490 nm. The concentration of total soluble sugar was expressed as µg/gm fw. The experiment was conducted in triplicate for each set.

### Metabolomics study

The metabolite extraction and derivatisation were performed as described in a previous study^81^. Approximately 300 mg of leaves from both non-infected and infected [24 and 48 hpi] transgenic and WT rice plants were homogenised in 100% methanol (1.4 mL), into which 50 μL internal standard (2 mg/mL sorbitol) was added. The mixture was thoroughly vortexed, incubated in water bath for 15 min at 70 °C, and centrifuged at 8000 × g for 10 min. The methanol/water fraction (1000 μL) of the solution was vacuum dried in a speed vac and stored at −20 °C. 40 μL methoxyamine hydrochloride (20 mg/mL in pyridine) solutions was added to the dried samples and incubated for 90 min at 37 °C. Trimethylsilylation of the samples were performed by adding 60 μL MSTFA and incubated at 37 °C for 30 min. 1 µL of the prepared sample was injected in GC-MS (Shimadzu QP–2010) by using the autosampler. Three replicas for each sample were taken for metabolite analysis.

### Bioinformatics analysis

KEGG (Kyoto Encyclopedia of Genes and Genomes, www.kegg.jp/kegg/kegg1.html) was used^82^ for assigning the functional categories and hierarchies to the identified proteins. ExPASy (http://www.expasy.org/tools/pi_tool.html) was used to evaluate the theoretical peptide mass and pI of the polypeptides based on their corresponding positions in the 2-DE gel map^83,84^. A protein-protein interaction network was developed using the STRING version 10.5 software^85^ (available at http://string-db.org/) by entering the sequences of the identified proteins.

### Statistical analysis

GraphPad Prism 5 software (GraphPad Software, USA) was used for analysis of statistical data. Bonferroni Multiple Comparison Test and one-way and two-way analyses of variance (ANOVAs) were used to evaluate the differences between the non-transgenic WT control and the transgenic rice plants, respectively. Statistical significant was considered to be *P* < 0.05.

## Supplementary information

Supplementary information
