# Supplementary material for: Proteo-metabolomic investigation of transgenic rice unravels metabolic alterations and accumulation of novel proteins potentially involved in defence against Rhizoctonia solani
Source: Sci Rep. 2019 Jul 18;9:10461. doi: 10.1038/s41598-019-46885-3 (PMC6639406; doi:10.1038/s41598-019-46885-3)
Supplement: Supplementary file 1 — Supplementary information [file 41598_2019_46885_MOESM1_ESM.pdf]

Proteo-metabolomic investigation of transgenic rice unravels metabolic alterations and accumulation of novel proteins potentially involved in defence against *Rhizoctonia solani*

Subhasis Karmakar[1], Karabi Datta[1]*, Kutubuddin Ali Molla[2, 3], Dipak Gayen[4], Kaushik Das[1], Sailendra Nath Sarkar[1], and Swapan K. Datta[1]

*(Corresponding author)

Dept. of Botany, University of Calcutta, 35 Ballygunge Circular Road, Kolkata- 700019

Email ID: krbdatta@yahoo.com

Ph No: +91339830538205

Fax no: +9133 2461 4849

Supplementary Fig S1. Diagrammatic representation of gene constructs and molecular evaluation transgenic rice lines harbouring *AtNPR1* gene. (a) Schematic representation of T-DNA construct harboring *AtNPR1* gene under CaMV35s promoter for rice transformation. (b) PCR analysis of transgenic rice plants with partial gene-specific (*AtNPR1*) primers which amplified 1.7Kbp product. (c) Relative quantity of rice *AtNPR1* mRNA in leaves of transgenic and WT rice lines as determined by RT-PCR (Real time PCR). Result is the mean ± standard error (SE) of three independent experiments. (d) Semi quantitative RT-PCR analysis of selected *AtNPR1* rice lines using gene specific primers taking *β-tubulin* as reference control. (e) Immunoblot analysis showing expression of 66 kDa NPR1 protein in transgenic plants but absent in WT. (f) Southern blot analysis: genomic DNA digested with *Not*I restriction enzyme and probed with 1.1 kbp *NPR1* gene fragment. WT represents wild type PC-Positive control and NC-Negative control.

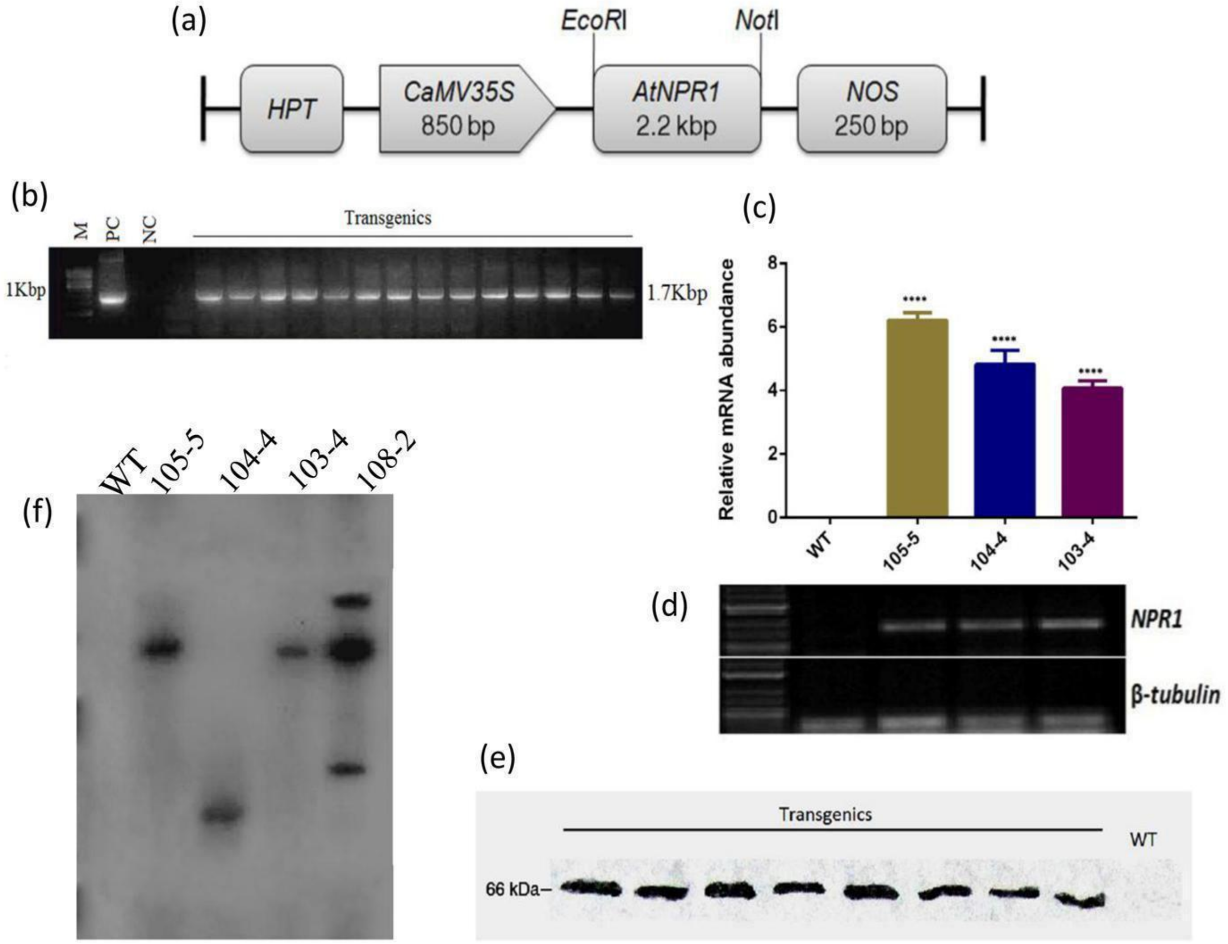

Supplementary Fig S2. Evaluation of transgenic rice lines against sheath blight disease along with non-transformed WT (Wild type) through *In vitro* and *In vivo* plant bioassay. (a) Bar diagram showing reduced infection cushion formation in transgenic lines than in the wild-type (WT). Experiments replicated three times. (b) Representative images of reduced lesion formation in transgenic leaves relative to WT in mycellial agar disc bioassay. (c) Bar diagram showing higher percentage of remaining green area after 72 hrs post infection in transgenic leaf samples than wild type (WT) in RS -toxin bioassay. (d) Images showing less affected area in transgenic leaves compared to WT control in the toxin bioassay. (e) Percent Disease Index (PDI) value in transgenic plants in respect to wild type control at 7, 14 and 21 dpi (days post inoculation). The values represent as the mean ± SE (n = 15). (f) Representative images showing typical sheath blight symptoms development on rice tillers of transgenic and non transformed wild type IR-64 control. Pictures were taken at 21 dpi. Red arrows indicate sheath blight symptoms.

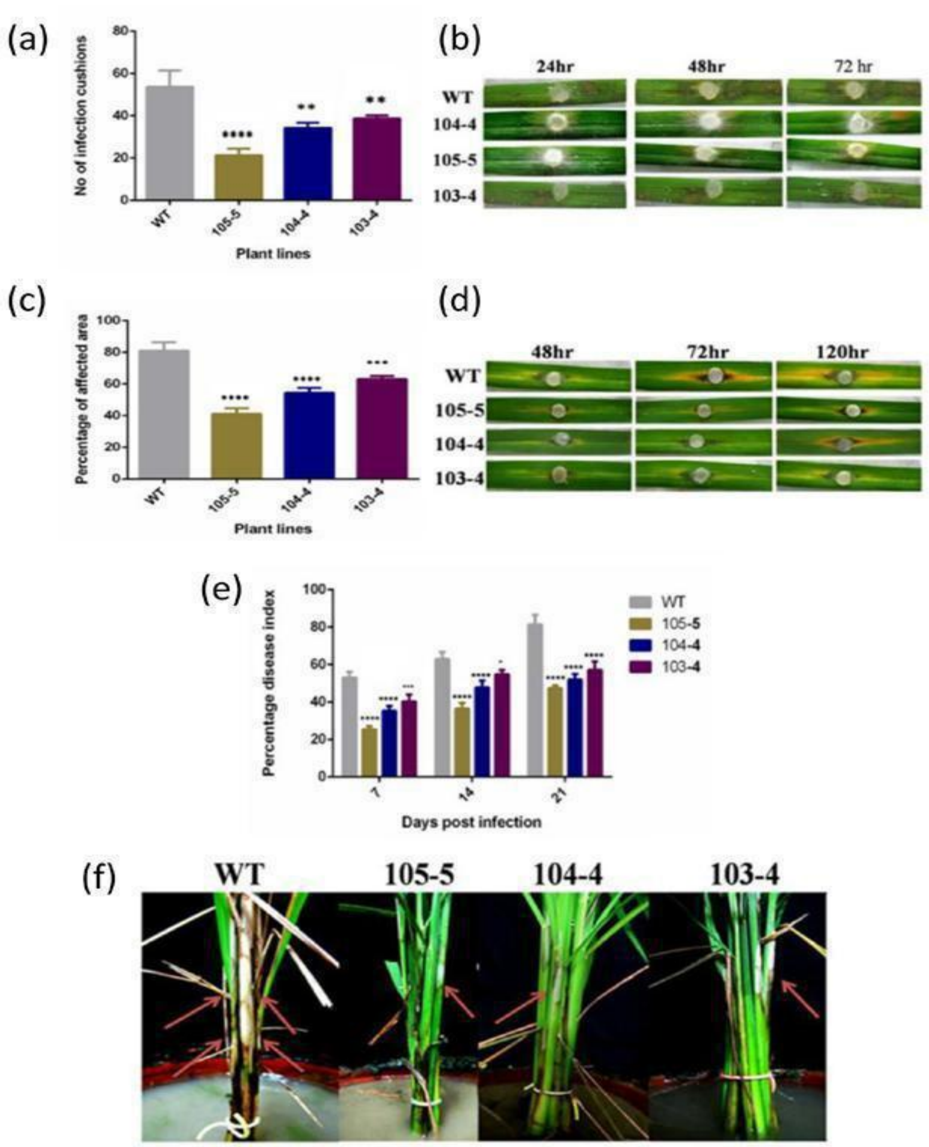

Supplementary Fig S3: Representative 2-DE replicated gel images of leaf proteins isolated from two sets of three plants (3 plants each from wild type and homozygous transgenic plants of 105-5 line) in three different time points [(BI): WT before infection, transgenic before infection, AI (24hr): WT after 24 hr infection, transgenic after 24 hr infection, AI (48 hr): WT after 48 hr infection, transgenic after 48 hr infection]. 800 µg of total protein from each sample was loaded on IPG strips (17 cm), linear pH 4–7 gradient, and separated by 12% SDS PAGE.

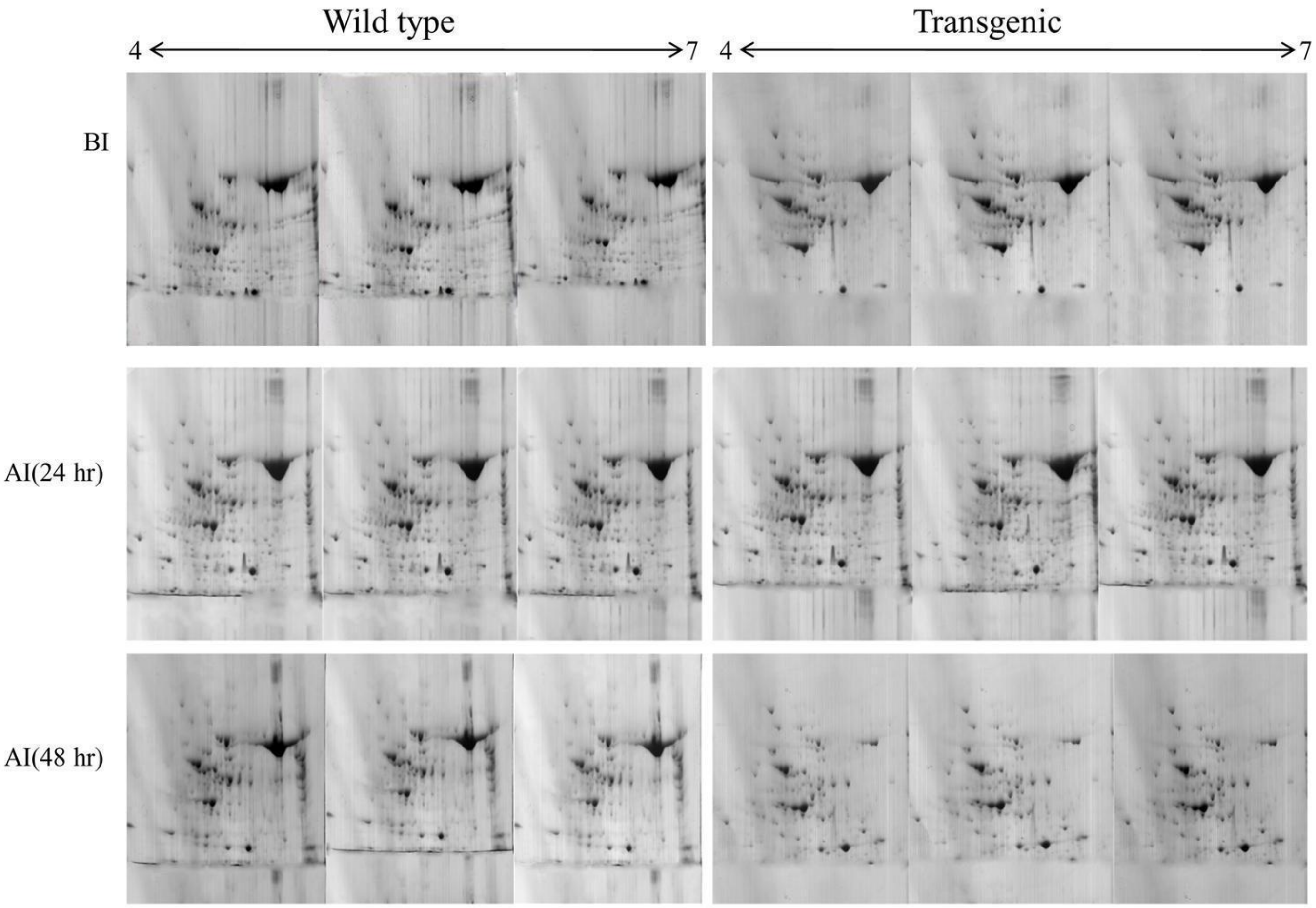

Supplementary Fig S4: Protein-protein interaction network analysis of identified proteins using STRING version 10.5 (available at http://string-db.org/)[80]. (a) Interactions of different high abundant protein groups including energy and primary metabolism related proteins, stress and defense related group of proteins, to other low abundant proteins. Different line colors signifies the types of evidence for each association: red line, fusion evidence; green line, neighborhood evidence; black line, co-expression evidence; purple line, experimental evidence; blue line, co-occurrence evidence; light blue line, database evidence; light purple line, homology evidence; and yellow line, text-mining evidence; (b) Interaction among energy and primary metabolism related (c) Interaction among stress and defense related proteins.

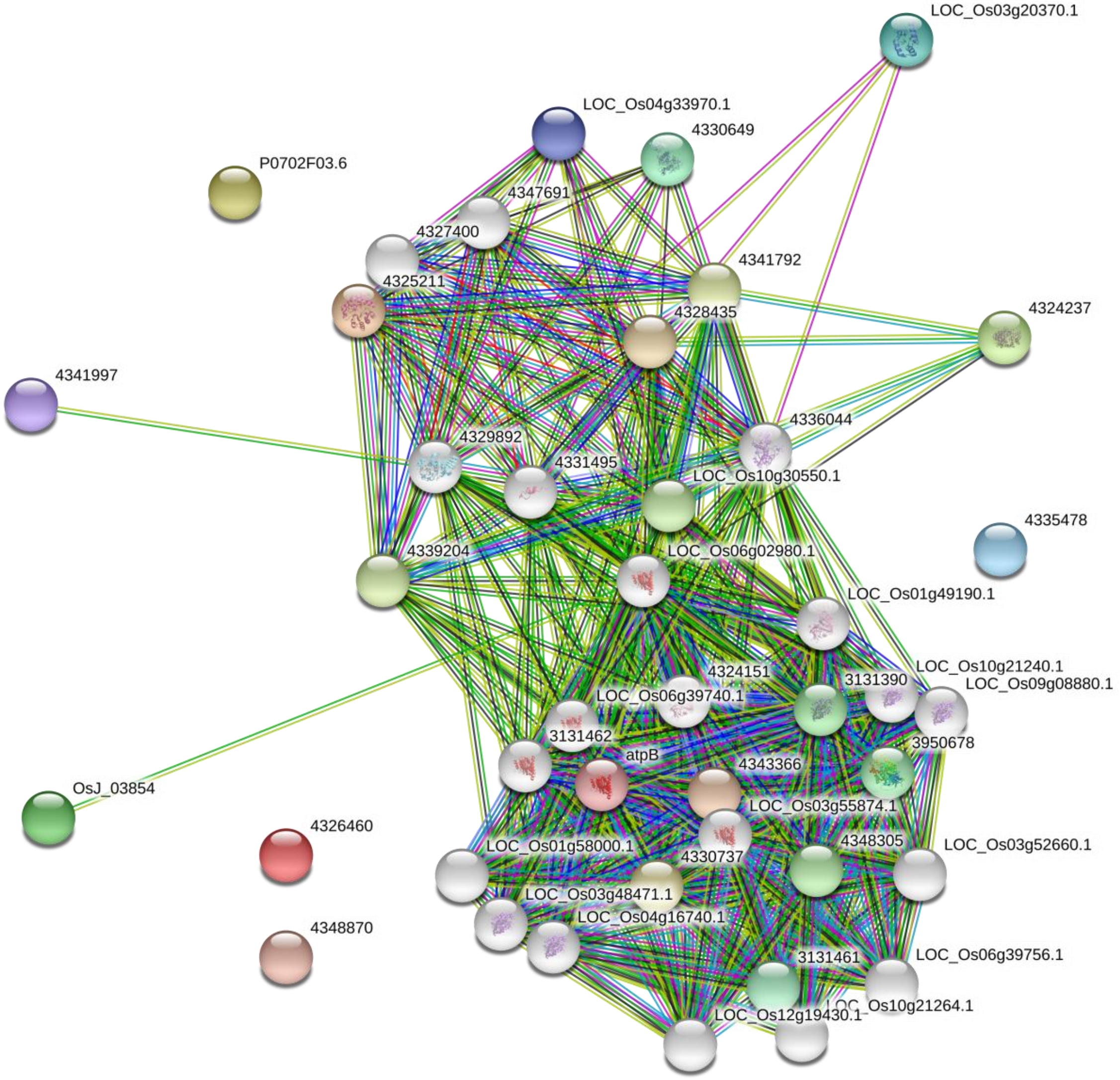

(a)

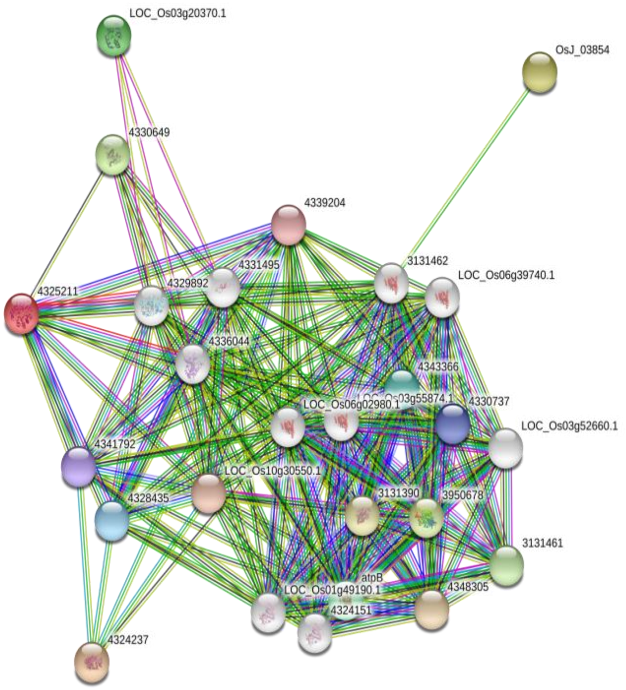

LOC_Os03g20370.1
OsJ_03854
4330649
4339204
4331495
3131462
LOC_Os06g39740.1
4325211
4329892
4336044
4343366
LOC_Os06g02980.1
4330737
LOC_Os03g52660.1
4341792
LOC_Os10g30550.1
4328435
3131390
3950678
3131461
atpB
LOC_Os01g49190.1
4348305
4324151
4324237

(b)

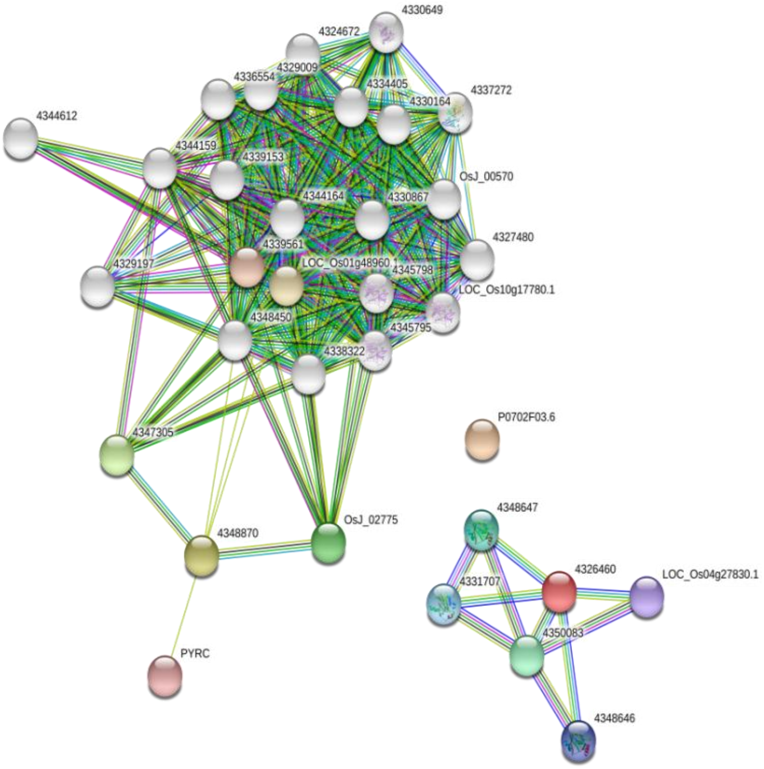

4330649
4324672
4329009
4336554
4334405
4337272
4330164
4344612
4344159
4339153
OsJ_00570
4344164
4330867
4327480
4339561
LOC_Os01g48960.1
4345798
LOC_Os10g17780.1
4329197
4348450
4345795
4338322
4347305
P0702F03.6
4348647
OsJ_02775
4348870
4326460
LOC_Os04g27830.1
4331707
4350083
PYRC
4348646

(c)

Supplementary Fig S5 Amount of total soluble sugar content in WT and transgenic *AtNPR1* overexpressing rice plants measured in three different time points [BI: Before infection, AI: After infection (24hr) and (48hr)]. The elevated amount of sugar content in transgenic plant after sheath blight pathostress in both time points represent an increment in carbohydrate metabolism. Data represent means ± SE calculated from three replicates.

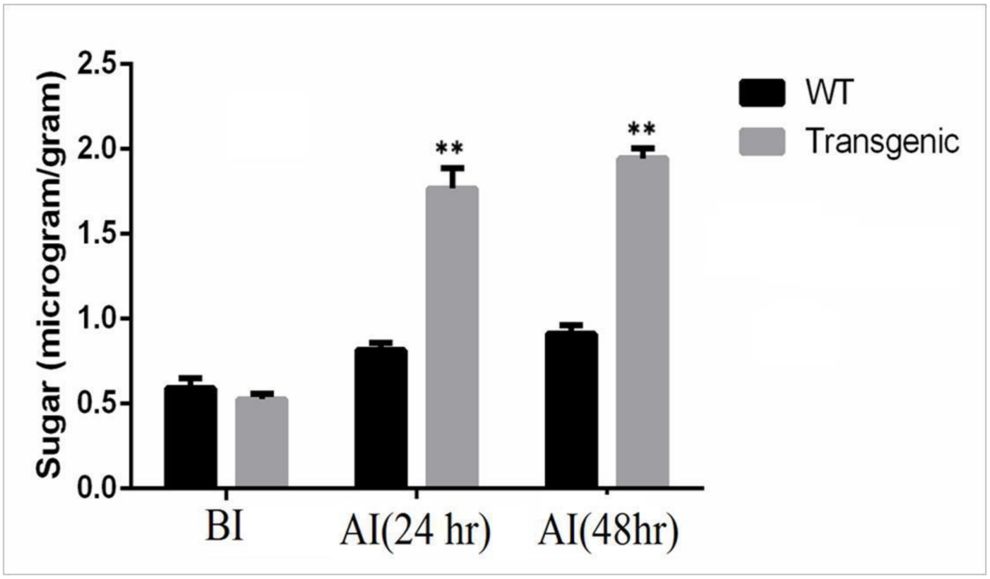

Supplementary Fig. S6

Several key enzymes identified through proteomic analysis were analyzed using KEGG (www.kegg.jp/kegg/kegg1.html)[77] metabolic pathway map. (a) Alpha- amino acid metabolism, (b) Glycolysis/Gluconeogenesis, (c)-(e) Lipid metabolism, (f) Drug metabolism, and (g) Cyanoamino acid metabolism. EC number with different colour indicates enzymes identified through proteomics participated in different metabolic pathways.

(a)

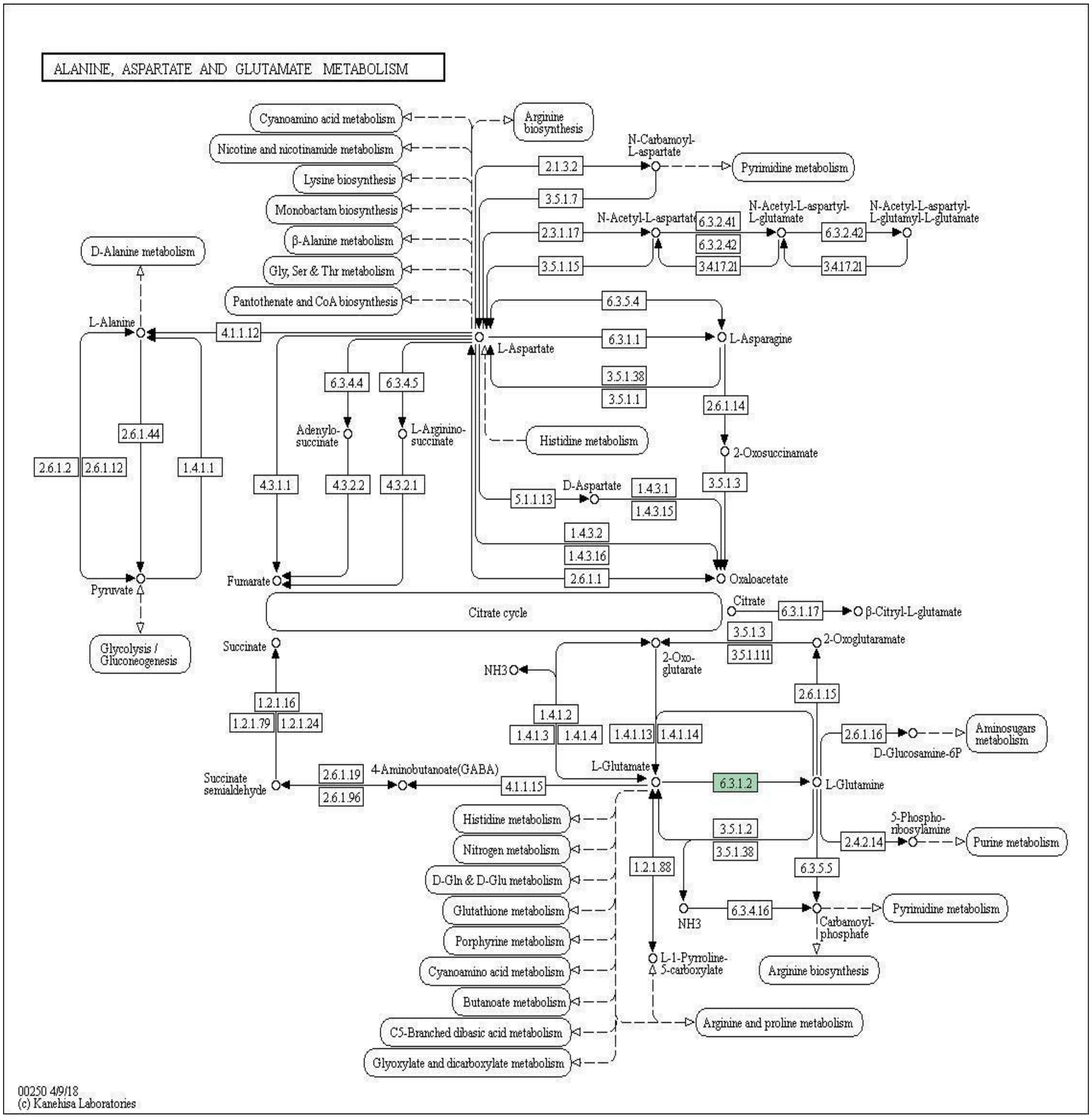

(b)

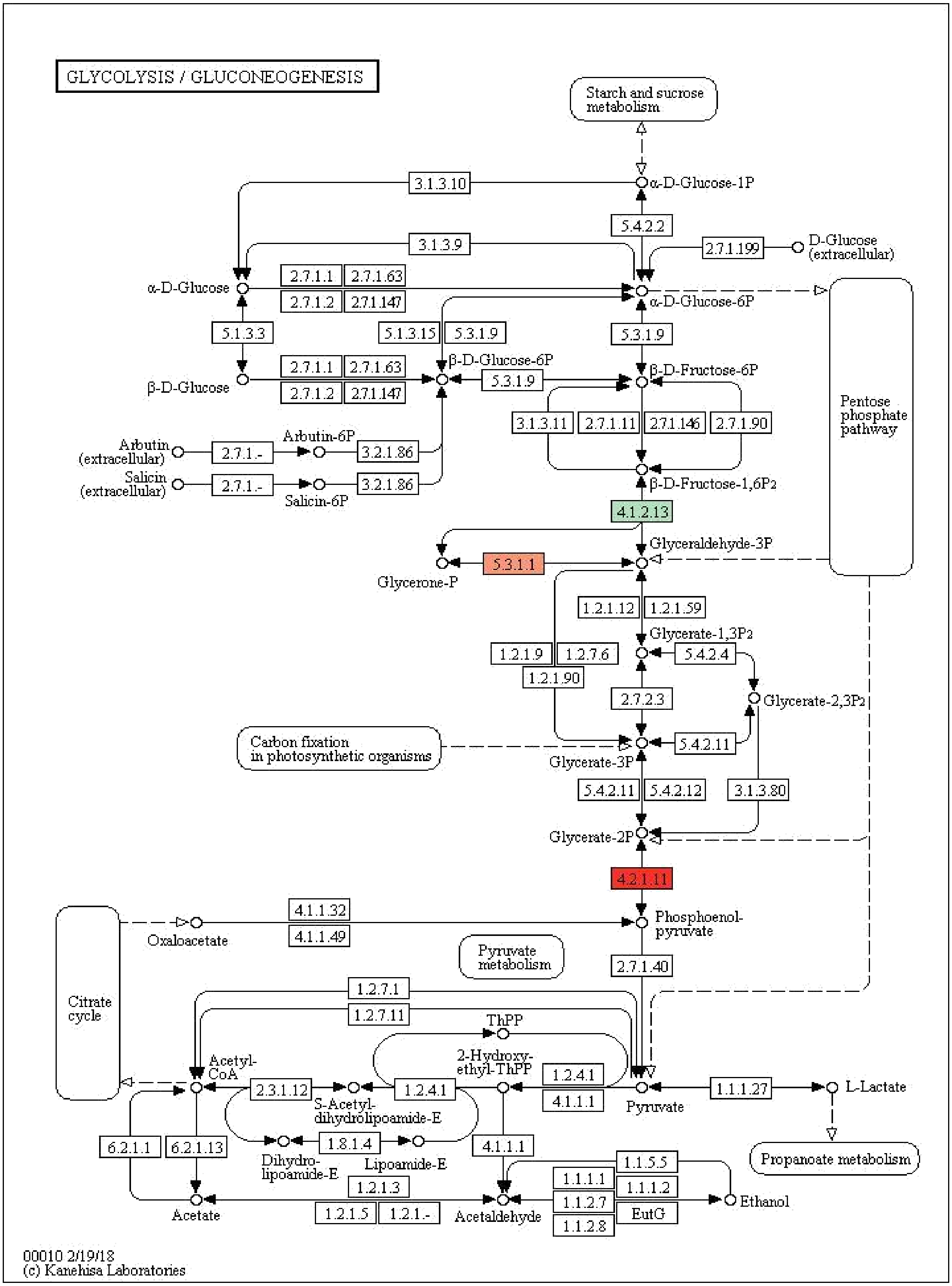
GLYCOLYSIS / GLUCONEOGENESIS
Starch and sucrose metabolism
α-D-Glucose-1P
3.1.3.10
5.4.2.2
3.1.3.9
2.7.1.199
D-Glucose (extracellular)
α-D-Glucose
2.7.1.1
2.7.1.63
2.7.1.2
2.7.1.147
α-D-Glucose-6P
5.1.3.3
5.1.3.15
5.3.1.9
β-D-Glucose
β-D-Glucose-6P
β-D-Fructose-6P
Pentose phosphate pathway
3.1.3.11
2.7.1.11
2.7.1.146
2.7.1.90
Arbutin (extracellular)
2.7.1.-
Arbutin-6P
3.2.1.86
Salicin (extracellular)
Salicin-6P
β-D-Fructose-1,6P2
4.1.2.13
Glyceraldehyde-3P
5.3.1.1
Glycerone-P
1.2.1.12
1.2.1.59
Glycerate-1,3P2
1.2.1.9
1.2.7.6
5.4.2.4
1.2.1.90
2.7.2.3
Glycerate-2,3P2
Carbon fixation in photosynthetic organisms
5.4.2.11
Glycerate-3P
5.4.2.12
3.1.3.80
Glycerate-2P
4.2.1.11
Citrate cycle
4.1.1.32
4.1.1.49
Oxaloacetate
Phosphoenol-pyruvate
Pyruvate metabolism
2.7.1.40
1.2.7.1
1.2.7.11
ThPP
Acetyl-CoA
2-Hydroxy-ethyl-ThPP
1.2.4.1
2.3.1.12
4.1.1.1
Pyruvate
1.1.1.27
L-Lactate
S-Acetyl-dihydrolipoamide-E
1.8.1.4
Dihydro-lipoamide-E
Lipoamide-E
6.2.1.1
6.2.1.13
1.1.5.5
Propanoate metabolism
1.1.1.1
1.1.1.2
1.2.1.3
1.1.2.7
Ethanol
Acetate
1.2.1.5
1.2.1.-
Acetaldehyde
EutG
1.1.2.8
00010 2/19/18
(c) Kanehisa Laboratories

(c)

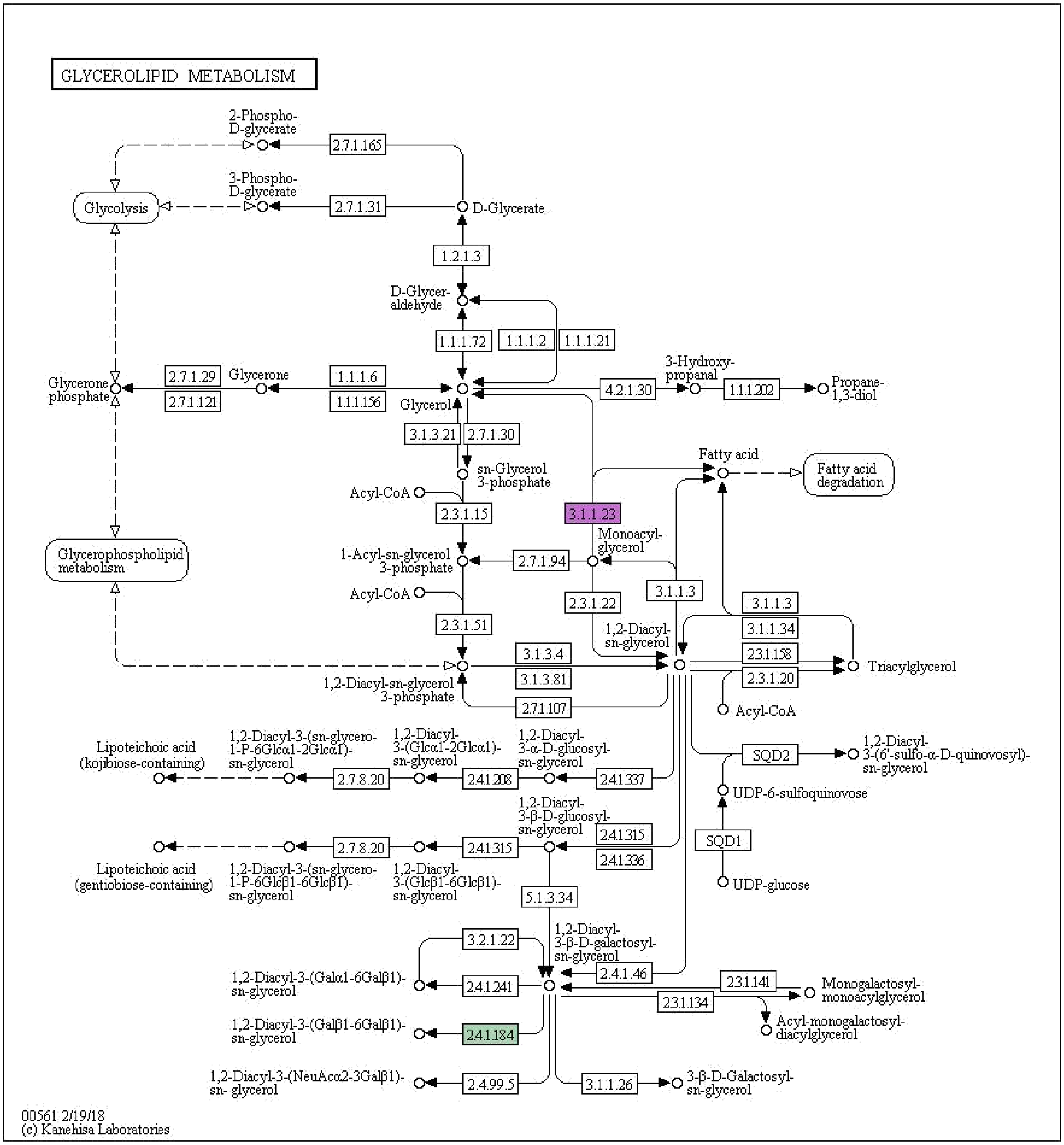

GLYCEROLIPID METABOLISM
2-Phospho-D-glycerate
2.7.1.165
3-Phospho-D-glycerate
2.7.1.31
Glycolysis
D-Glycerate
1.2.1.3
D-Glyceraldehyde
1.1.1.72
1.1.1.2
1.1.1.21
Glycerone phosphate
2.7.1.29
2.7.1.121
Glycerone
1.1.1.6
1.1.1.156
Glycerol
4.2.1.30
3-Hydroxy-propanal
1.1.1.202
Propane-1,3-diol
3.1.3.21
2.7.1.30
sn-Glycerol 3-phosphate
Acyl-CoA
2.3.1.15
3.1.1.23
Fatty acid
Fatty acid degradation
Glycerophospholipid metabolism
1-Acyl-sn-glycerol 3-phosphate
2.7.1.94
Monoacyl-glycerol
3.1.1.3
2.3.1.22
2.3.1.51
1,2-Diacyl-sn-glycerol
3.1.1.34
2.3.1.158
2.3.1.20
Triacylglycerol
3.1.3.4
3.1.3.81
2.7.1.107
1,2-Diacyl-sn-glycerol 3-phosphate
Acyl-CoA
Lipoteichoic acid (kojibiose-containing)
1,2-Diacyl-3-(sn-glycero-1-P-6Glcα1-2Glcα1)-sn-glycerol
1,2-Diacyl-3-(Glcα1-2Glcα1)-sn-glycerol
1,2-Diacyl-3-α-D-glucosyl-sn-glycerol
2.7.8.20
2.4.1.208
2.4.1.337
SQD2
1,2-Diacyl-3-(6'-sulfo-α-D-quinovosyl)-sn-glycerol
UDP-6-sulfoquinovose
1,2-Diacyl-3-β-D-glucosyl-sn-glycerol
2.4.1.315
2.4.1.336
SQD1
UDP-glucose
Lipoteichoic acid (gentiobiose-containing)
1,2-Diacyl-3-(sn-glycero-1-P-6Glcβ1-6Glcβ1)-sn-glycerol
1,2-Diacyl-3-(Glcβ1-6Glcβ1)-sn-glycerol
5.1.3.34
3.2.1.22
1,2-Diacyl-3-β-D-galactosyl-sn-glycerol
2.4.1.46
2.3.1.141
2.3.1.134
Monogalactosyl-monoacylglycerol
Acyl-monogalactosyl-diacylglycerol
1,2-Diacyl-3-(Galα1-6Galβ1)-sn-glycerol
2.4.1.241
1,2-Diacyl-3-(Galβ1-6Galβ1)-sn-glycerol
2.4.1.184
1,2-Diacyl-3-(NeuAcα2-3Galβ1)-sn- glycerol
2.4.99.5
3.1.1.26
3-β-D-Galactosyl-sn-glycerol
00561 2/19/18
(c) Kanehisa Laboratories

(d)

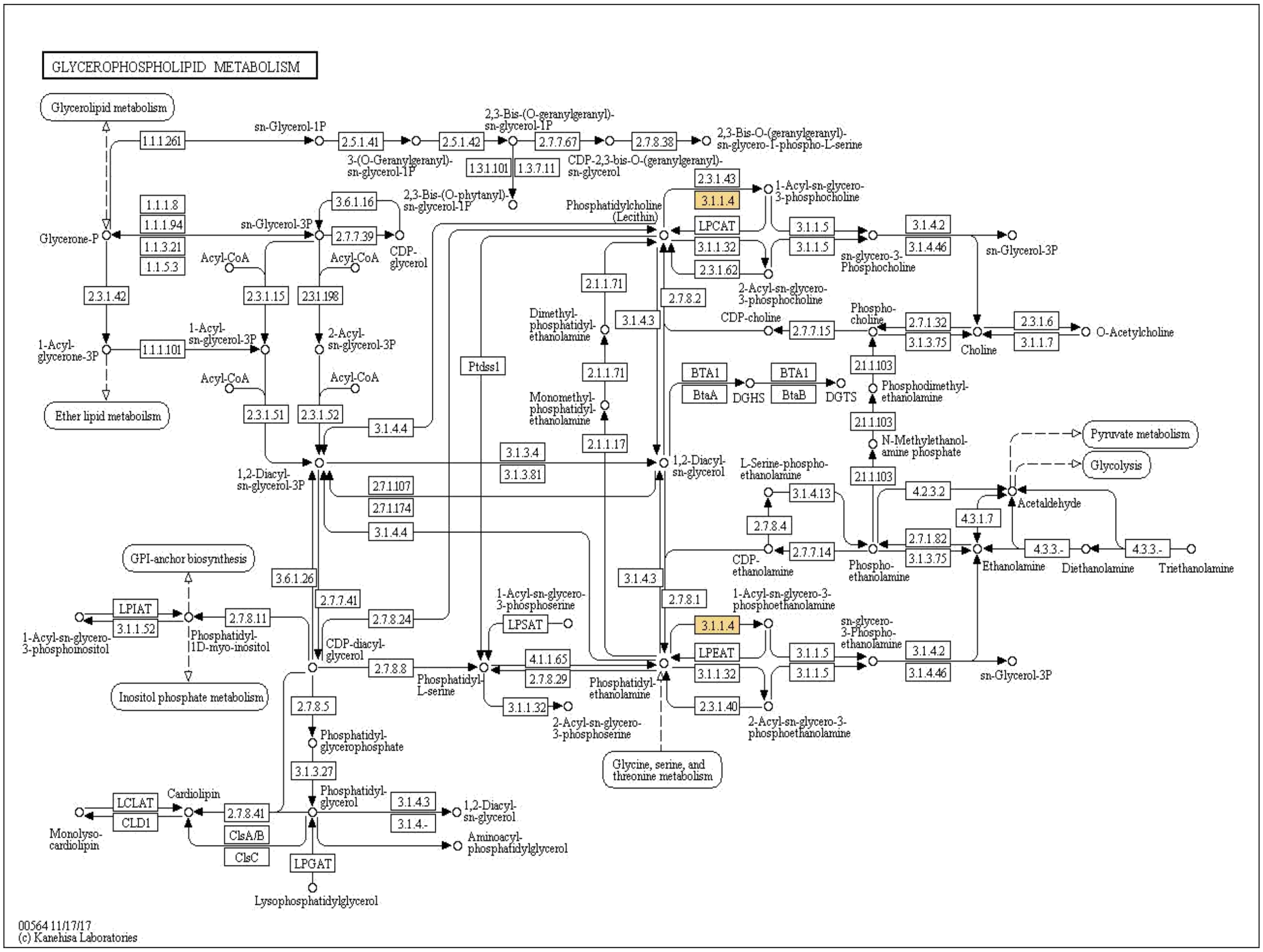
GLYCEROPHOSPHOLIPID METABOLISM
Glycerolipid metabolism
sn-Glycerol-1P
2,3-Bis-(O-geranylgeranyl)-sn-glycerol-1P
3-(O-Geranylgeranyl)-sn-glycerol-1P
CDP-2,3-bis-O-(geranylgeranyl)-sn-glycerol
2,3-Bis-O-(geranylgeranyl)-sn-glycero-1-phospho-L-serine
2,3-Bis-(O-phytanyl)-sn-glycerol-1P
Glycerone-P
sn-Glycerol-3P
CDP-glycerol
Acyl-CoA
1-Acyl-glycerone-3P
1-Acyl-sn-glycerol-3P
2-Acyl-sn-glycerol-3P
Ether lipid metabolism
1,2-Diacyl-sn-glycerol-3P
Phosphatidylcholine (Lecithin)
1-Acyl-sn-glycero-3-phosphocholine
sn-glycero-3-Phosphocholine
2-Acyl-sn-glycero-3-phosphocholine
sn-Glycerol-3P
Dimethyl-phosphatidyl-ethanolamine
Monomethyl-phosphatidyl-ethanolamine
CDP-choline
Phospho-choline
Choline
O-Acetylcholine
DGHS
DGTS
Phosphodimethyl-ethanolamine
N-Methylethanol-amine phosphate
Pyruvate metabolism
Glycolysis
Acetaldehyde
1,2-Diacyl-sn-glycerol
L-Serine-phospho-ethanolamine
CDP-ethanolamine
Phospho-ethanolamine
Ethanolamine
Diethanolamine
Triethanolamine
GPI-anchor biosynthesis
1-Acyl-sn-glycero-3-phosphoinositol
Phosphatidyl-1D-myo-inositol
Inositol phosphate metabolism
CDP-diacyl-glycerol
1-Acyl-sn-glycero-3-phosphoserine
Phosphatidyl-L-serine
1-Acyl-sn-glycero-3-phosphoethanolamine
Phosphatidyl-ethanolamine
sn-glycero-3-Phospho-ethanolamine
2-Acyl-sn-glycero-3-phosphoserine
2-Acyl-sn-glycero-3-phosphoethanolamine
Glycine, serine, and threonine metabolism
Phosphatidyl-glycerophosphate
Phosphatidyl-glycerol
Cardiolipin
Monolyso-cardiolipin
1,2-Diacyl-sn-glycerol
Aminoacyl-phosphatidylglycerol
Lysophosphatidylglycerol
1.1.1.261
2.5.1.41
2.5.1.42
2.7.7.67
2.7.8.38
1.3.1.101
1.3.7.11
1.1.1.8
1.1.1.94
1.1.3.21
1.1.5.3
3.6.1.16
2.7.7.39
2.3.1.42
2.3.1.15
2.3.1.198
1.1.1.101
2.3.1.51
2.3.1.52
3.1.4.4
2.3.1.43
3.1.1.4
LPCAT
3.1.1.32
2.3.1.62
3.1.1.5
3.1.4.2
3.1.4.46
2.1.1.71
2.7.8.2
3.1.4.3
2.7.7.15
2.7.1.32
3.1.3.75
2.3.1.6
3.1.1.7
Ptdss1
BTA1
BtaA
BtaB
2.1.1.103
2.1.1.17
3.1.3.4
3.1.3.81
2.7.1.107
2.7.1.174
3.1.4.13
4.2.3.2
4.3.1.7
2.7.8.4
2.7.7.14
2.7.1.82
4.3.3.-
3.6.1.26
2.7.7.41
2.7.8.24
LPIAT
3.1.1.52
2.7.8.11
2.7.8.1
LPSAT
LPEAT
4.1.1.65
2.7.8.29
2.7.8.8
2.7.8.5
2.3.1.40
3.1.3.27
LCLAT
CLD1
2.7.8.41
ClsA/B
ClsC
LPGAT
3.1.4.-
00564 11/17/17
(c) Kanehisa Laboratories

(e)

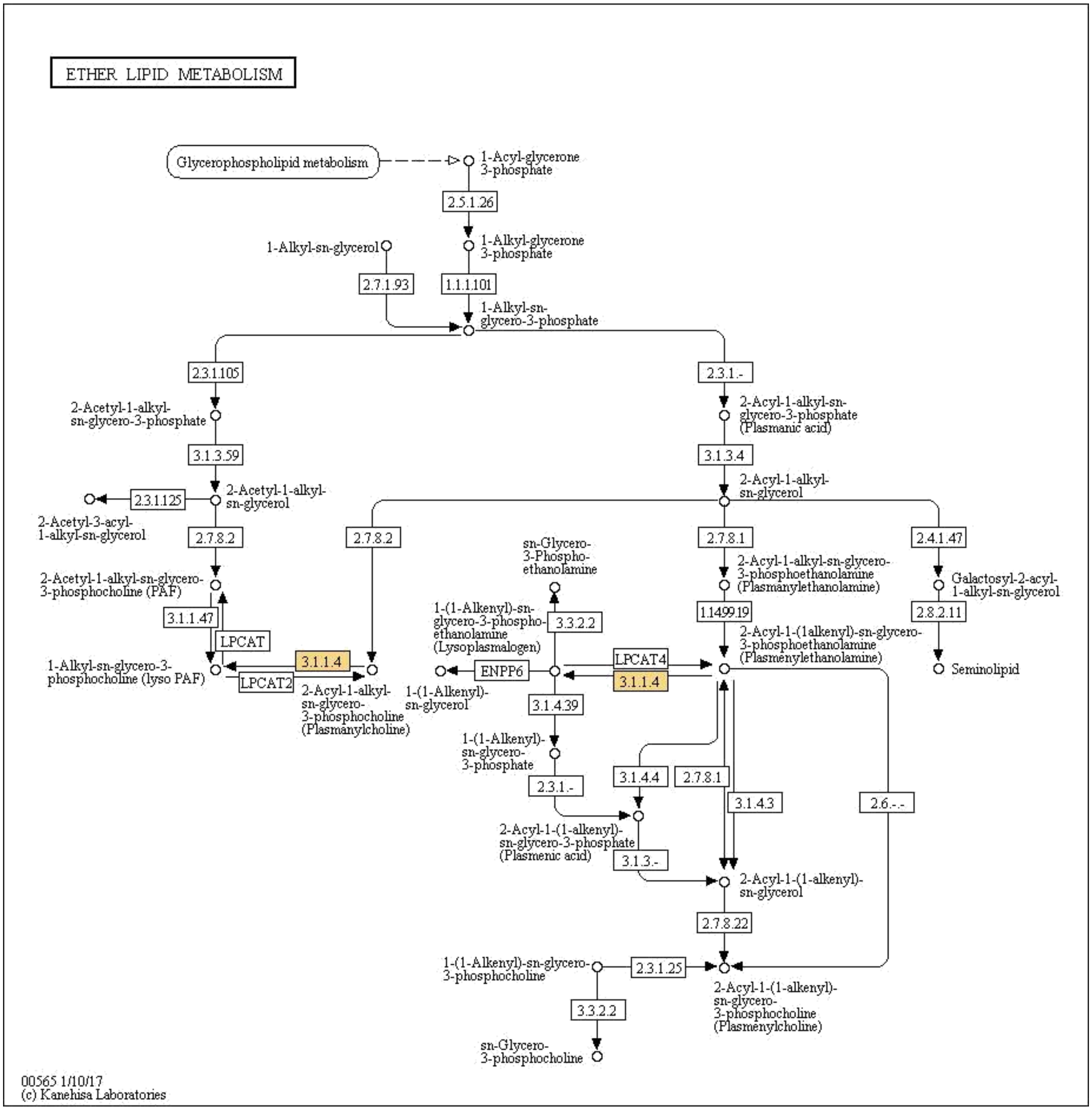
ETHER LIPID METABOLISM
Glycerophospholipid metabolism
1-Acyl-glycerone 3-phosphate
2.5.1.26
1-Alkyl-glycerone 3-phosphate
1-Alkyl-sn-glycerol
2.7.1.93
1.1.1.101
1-Alkyl-sn-glycero-3-phosphate
2.3.1.105
2.3.1.-
2-Acetyl-1-alkyl-sn-glycero-3-phosphate
2-Acyl-1-alkyl-sn-glycero-3-phosphate (Plasmanic acid)
3.1.3.59
3.1.3.4
2-Acetyl-1-alkyl-sn-glycerol
2-Acyl-1-alkyl-sn-glycerol
2.3.1.125
2-Acetyl-3-acyl-1-alkyl-sn-glycerol
2.7.8.2
2.7.8.2
2.7.8.1
2.4.1.47
2-Acetyl-1-alkyl-sn-glycero-3-phosphocholine (PAF)
sn-Glycero-3-Phospho-ethanolamine
2-Acyl-1-alkyl-sn-glycero-3-phosphoethanolamine (Plasmanylethanolamine)
Galactosyl-2-acyl-1-alkyl-sn-glycerol
3.1.1.47
LPCAT
1-(1-Alkenyl)-sn-glycero-3-phospho-ethanolamine (Lysoplasmalogen)
3.3.2.2
1.14.99.19
2.8.2.11
2-Acyl-1-(1alkenyl)-sn-glycero-3-phosphoethanolamine (Plasmenylethanolamine)
3.1.1.4
LPCAT4
1-Alkyl-sn-glycero-3-phosphocholine (lyso PAF)
LPCAT2
ENPP6
3.1.1.4
Seminolipid
2-Acyl-1-alkyl-sn-glycero-3-phosphocholine (Plasmanylcholine)
1-(1-Alkenyl)-sn-glycerol
3.1.4.39
1-(1-Alkenyl)-sn-glycero-3-phosphate
3.1.4.4
2.7.8.1
2.3.1.-
3.1.4.3
2.6.-.-
2-Acyl-1-(1-alkenyl)-sn-glycero-3-phosphate (Plasmenic acid)
3.1.3.-
2-Acyl-1-(1-alkenyl)-sn-glycerol
2.7.8.22
1-(1-Alkenyl)-sn-glycero-3-phosphocholine
2.3.1.25
2-Acyl-1-(1-alkenyl)-sn-glycero-3-phosphocholine (Plasmenylcholine)
3.3.2.2
sn-Glycero-3-phosphocholine
00565 1/10/17
(c) Kanehisa Laboratories

(f)

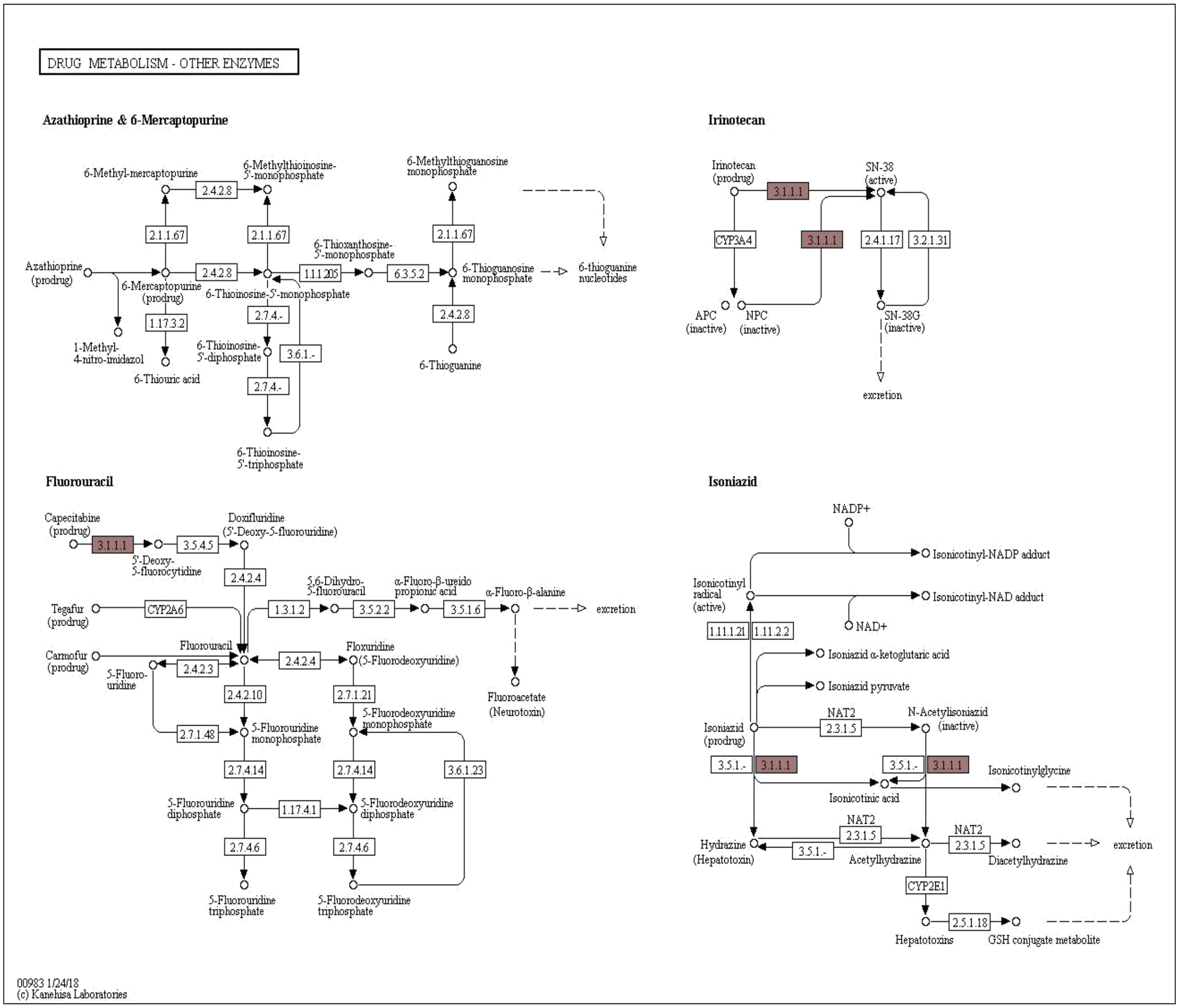
DRUG METABOLISM - OTHER ENZYMES
Azathioprine & 6-Mercaptopurine
6-Methyl-mercaptopurine
6-Methylthioinosine-5'-monophosphate
6-Methylthioguanosine monophosphate
Azathioprine (prodrug)
6-Mercaptopurine (prodrug)
6-Thioinosine-5'-monophosphate
6-Thioxanthosine-5'-monophosphate
6-Thioguanosine monophosphate
6-thioguanine nucleotides
1-Methyl-4-nitro-imidazol
6-Thiouric acid
6-Thioinosine-5'-diphosphate
6-Thioinosine-5'-triphosphate
6-Thioguanine
2.4.2.8
2.1.1.67
1.17.3.2
2.7.4.-
3.6.1.-
1.1.1.205
6.3.5.2
Irinotecan
Irinotecan (prodrug)
SN-38 (active)
3.1.1.1
CYP3A4
2.4.1.17
3.2.1.31
APC (inactive)
NPC (inactive)
SN-38G (inactive)
excretion
Fluorouracil
Capecitabine (prodrug)
Doxifluridine (5'-Deoxy-5-fluorouridine)
5'-Deoxy-5-fluorocytidine
3.5.4.5
2.4.2.4
Tegafur (prodrug)
CYP2A6
5,6-Dihydro-5-fluorouracil
α-Fluoro-β-ureido propionic acid
α-Fluoro-β-alanine
1.3.1.2
3.5.2.2
3.5.1.6
excretion
Carmofur (prodrug)
Fluorouracil
Floxuridine (5-Fluorodeoxyuridine)
5-Fluoro-uridine
2.4.2.3
2.4.2.10
2.7.1.21
2.7.1.48
Fluoroacetate (Neurotoxin)
5-Fluorouridine monophosphate
5-Fluorodeoxyuridine monophosphate
2.7.4.14
3.6.1.23
5-Fluorouridine diphosphate
1.17.4.1
5-Fluorodeoxyuridine diphosphate
2.7.4.6
5-Fluorouridine triphosphate
5-Fluorodeoxyuridine triphosphate
Isoniazid
NADP+
Isonicotinyl-NADP adduct
Isonicotinyl radical (active)
Isonicotinyl-NAD adduct
NAD+
1.11.1.21
1.11.2.2
Isoniazid α-ketoglutaric acid
Isoniazid pyruvate
Isoniazid (prodrug)
NAT2
2.3.1.5
N-Acetylisoniazid (inactive)
3.5.1.-
3.1.1.1
Isonicotinylglycine
Isonicotinic acid
Hydrazine (Hepatotoxin)
Acetylhydrazine
Diacetylhydrazine
excretion
CYP2E1
2.5.1.18
Hepatotoxins
GSH conjugate metabolite
00983 1/24/18
(c) Kanehisa Laboratories

(g)

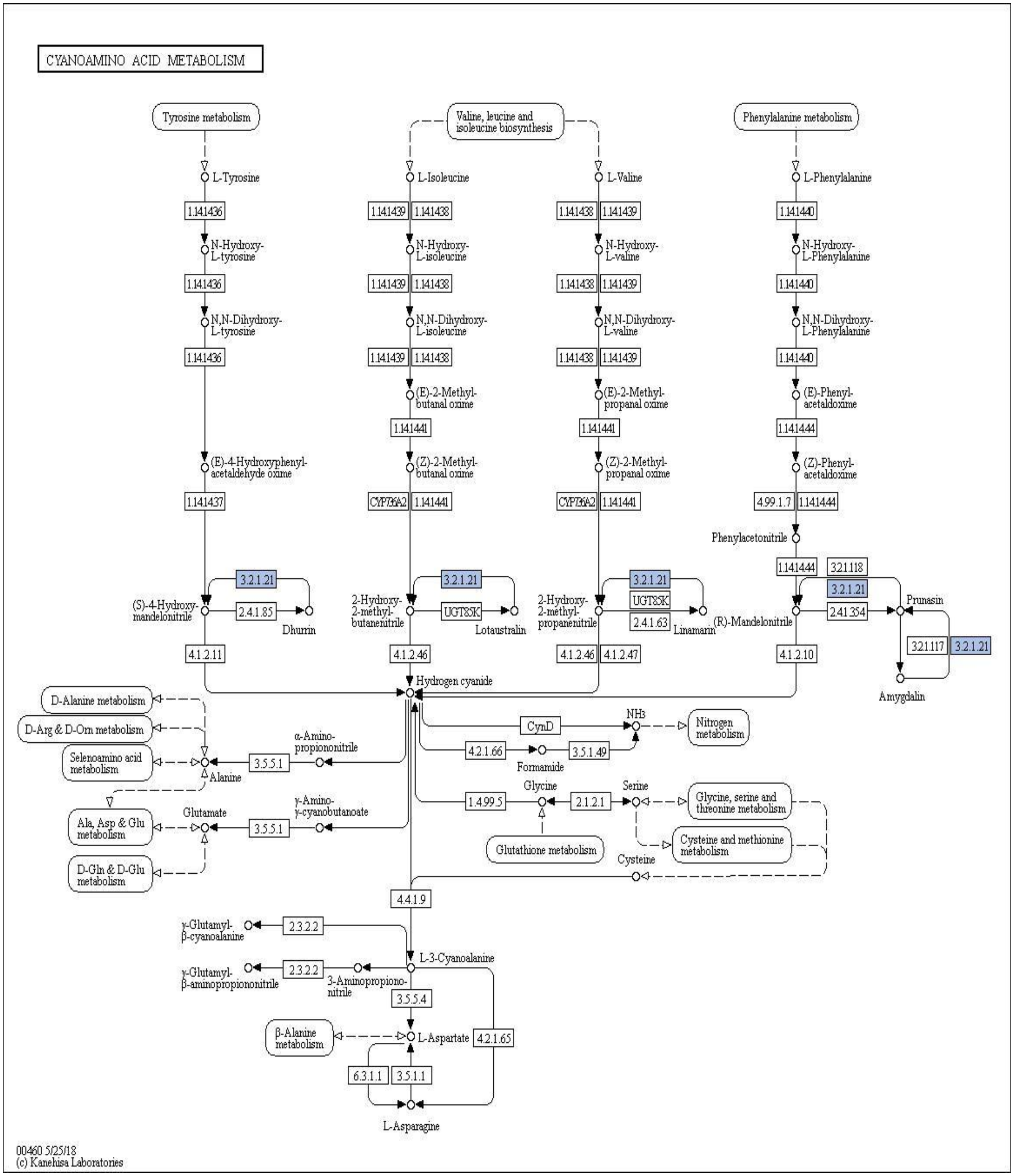
CYANOAMINO ACID METABOLISM
Tyrosine metabolism
Valine, leucine and isoleucine biosynthesis
Phenylalanine metabolism
L-Tyrosine
L-Isoleucine
L-Valine
L-Phenylalanine
1.14.14.36
1.14.14.39
1.14.14.38
1.14.14.40
N-Hydroxy-L-tyrosine
N-Hydroxy-L-isoleucine
N-Hydroxy-L-valine
N-Hydroxy-L-Phenylalanine
N,N-Dihydroxy-L-tyrosine
N,N-Dihydroxy-L-isoleucine
N,N-Dihydroxy-L-valine
N,N-Dihydroxy-L-Phenylalanine
(E)-2-Methylbutanal oxime
(E)-2-Methylpropanal oxime
(E)-Phenylacetaldoxime
1.14.14.41
1.14.14.44
(E)-4-Hydroxyphenylacetaldehyde oxime
(Z)-2-Methylbutanal oxime
(Z)-2-Methylpropanal oxime
(Z)-Phenylacetaldoxime
1.14.14.37
CYP736A2
4.99.1.7
Phenylacetonitrile
3.2.1.118
3.2.1.21
(S)-4-Hydroxymandelonitrile
2.4.1.85
Dhurrin
2-Hydroxy-2-methylbutanenitrile
UGT85K
Lotaustralin
2-Hydroxy-2-methylpropanenitrile
2.4.1.63
Linamarin
(R)-Mandelonitrile
2.4.1.354
Prunasin
3.2.1.117
Amygdalin
4.1.2.11
4.1.2.46
4.1.2.47
4.1.2.10
Hydrogen cyanide
D-Alanine metabolism
D-Arg & D-Orn metabolism
Selenoamino acid metabolism
Alanine
3.5.5.1
α-Amino-propiononitrile
Ala, Asp & Glu metabolism
Glutamate
γ-Amino-γ-cyanobutanoate
D-Gln & D-Glu metabolism
CynD
NH3
Nitrogen metabolism
4.2.1.66
Formamide
3.5.1.49
Glycine
Serine
1.4.99.5
2.1.2.1
Glycine, serine and threonine metabolism
Glutathione metabolism
Cysteine and methionine metabolism
Cysteine
4.4.1.9
γ-Glutamyl-β-cyanoalanine
2.3.2.2
L-3-Cyanoalanine
γ-Glutamyl-β-aminopropiononitrile
3-Aminopropiononitrile
3.5.5.4
β-Alanine metabolism
L-Aspartate
4.2.1.65
6.3.1.1
3.5.1.1
L-Asparagine
00460 5/25/18
(c) Kanehisa Laboratories

Table S1: Details of primers used for qRT-PCR analysis

Gene ID	Primers (5' to 3')	
	**Sense**	**Anti-sense**
OsBG (GI: 433213)	CCAAGGGGCTGTATGTATGG	GATGGACCCTGCACATTACC
OsDOF (GI: 1443091522)	ATGCCAAGGTTGGAGAGAGA	CAACCCCAACTCCAAGCTAA
OsNAC6 (GI:13272280)	TGCGCACAGTTCTCTTCAGT	ATCAAGGTCACCCGGATCAT
OsCAPD (GI: 1443087711)	GACAGGAAATCTCTTGTTTGGTTT	TAGTAGTACTGTTGTCTGAAAGGCTAA
OsMADS (GI: 2961436)	CCTACAGCTGGTGGAGAAGC	AAGCCTCCTTAGCCGTTGAT
OsLOX (GI: 18448902)	CTCCATCAAGGAGTGGGTGT	ATGGTGGTCAGAACCTCGAC
OsEIN2 (GI: 37622420)	TGGGTACATTGACCTCGGGA	CAATGCAAGCCGCGAGATAC
OsAOS2 (GI:4332121)	AAGAAGGGGGAGATGCTGTT	TGCTTGTTGTCAACGCTAGG
OsMAPK6 (GI: 38146077)	TATGTGCACTCGGCAAATGT	TCGATACCAACGAGTGACCA
OsMAPK4 (GI: 4341956)	TCCAACCTCCTCATCGACTC	ACCTGCCCATGTAGAACTCG
Osβ-tubulin (GI: 508575)	GGAGTCACATGCTGCCTAAGGTT	TCACTGCCAGCTTACGGAGG

Table S2: Details of primers used for validation of proteomic data by qRT-PCR

Gene	Primers (5' to 3')	
	**Sense**	**Anti-sense**
2Cys-pex (2Cys-peroxiredoxin)	CGAGAAGTTGAACACTGAGA	GATCAAGACACCAAAGGACT
1Cys-pex (1Cys-peroxiredoxin)	ACACCTATGTCATCCTCTTCT	CGGTTCCCAGGCTTGT
FBP (Fructose bis-phosphatase aldolase)	CTTCACGTCAAGGACTACAA	CACAGGTTCAGATTATTGCG
TPI (Triose phosphate isomerease)	GGCGAATCAAGTGATTTTGT	TGCAATAGCTTTAGTTTGCG
14-3-3GF-14f	AGGACATTGCTTTGGCTGAG	TTACTGCCCCTCGCTGGAG
PP2C1 (Protein phosphatase 2C1)	GCCGCAGCTCCGACAA	CTACAGCATCAGCTGGGTGACA
